# MALDI-TOF MS Classification of Formosan Thraustochytrids and Their Use in Shrimp Farming

**DOI:** 10.1021/acs.jafc.5c08939

**Published:** 2026-03-30

**Authors:** Setya Widi Ayuning Permanasari, Hsiu-Chin Lin, Jia-Ying Lin, Mao-Xuan Hong, Hsin-Yang Chang, Wen-Di He, Li-Hua Lo, Yu-Liang Yang, Tse-Min Lee, Chih-Chuang Liaw

**Affiliations:** † Department of Marine Biotechnology and Resources, 34874 National Sun Yat-sen University, 70, Lien-Hai Rd., Kaohsiung 80424, Taiwan; ‡ Department of Fisheries and Marine Resources Management, Faculty of Fisheries and Marine Science, Brawijaya University, Veteran Rd, Malang, East Java, 65145 Indonesia; § Department of Life Sciences and Institute of Genome Sciences, National Yang-Ming Chiao Tung University, Taipei 11221, Taiwan; ∥ 38017 Agricultural Biotechnology Research Center, Academia Sinica, Taipei 115, Taiwan; ⊥ Graduate Institute of Natural Products, Kaohsiung Medical University, Kaohsiung 807, Taiwan; # Graduate Institute of Pharmacognosy, Taipei Medical University, Taipei 110, Taiwan

**Keywords:** MALDI-TOF MS Biotyper, thraustochytrids, ω-3 polyunsaturated fatty acids, docosahexaenoic acid, astaxanthin, *Schizochytrium*, *Aurantiochytrium*, shrimp aquaculture

## Abstract

Thraustochytrids are emerging marine protists for the sustainable production of high-value bioactives, particularly ω-3 polyunsaturated fatty acids (PUFAs) such as docosahexaenoic acid (DHA) and carotenoids like astaxanthin. We established a MALDI-TOF MS Biotyper database for 33 Formosan thraustochytrid strains isolated from Taiwanese mangroves and compared it with 18S rRNA gene-based phylogenetic analysis. Biotyper-based classification provided enhanced taxonomic resolution and, when integrated with PCA and K-means clustering of biochemical traits, enabled functional grouping of the strains. HPLC quantification revealed diverse DHA and astaxanthin yields, leading to the selection of two strains for *Litopenaeus vannamei* aquaculture trials: DHA-rich *Schizochytrium* sp. W1.2 and astaxanthin-rich *Aurantiochytrium* sp. YA7.1. W1.2 enhanced postlarval survival and growth, while YA7.1 improved feed conversion and adult size. These findings highlight the usefulness of integrated biochemical and proteomic classification to select strains effectively. They also reveal the dual benefits of thraustochytrids in microbial taxonomy and functional nutrition, supporting sustainable shrimp aquaculture.

## Graphical abstract

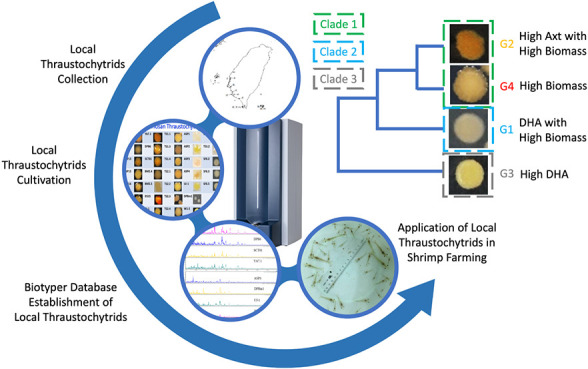

## Introduction

The global aquaculture industry is experiencing rapid growth, leading to an increasing demand for sustainable and efficient sources of essential nutritional supplements. Among these, ω-3 polyunsaturated fatty acids (PUFAs), especially docosahexaenoic acid (DHA, 22:6 n-3), and natural carotenoids, such as astaxanthin, have become increasingly vital. These compounds are widely recognized for their ability to enhance growth, boost immune response, and improve pigmentation in aquaculture species.^1,2^ Marine microorganisms are emerging as promising, renewable sources of these bioactive compounds.^3^ Various taxa, including bacteria such as *Shewanella* spp. and *Colwellia* spp.,^4^ yeasts like *Rhodotorula* spp.,^5^ and dinoflagellates including *Crypthecodinium cohnii* and *Isochrysis galbana*,^6,7^ have shown potential in their ability to produce PUFAs.^8^ Carotenoids play a crucial role in strengthening immune function and resistance to oxidative stress,^9,10^ and they are also utilized to promote pigmentation and growth in species such as salmon,^11^ shrimp,^12^ and ornamental fish.^12,13^ Particularly, *Phaffia rhodozyma* ^14^ and *Haematococcus pluvialis* are widely recognized natural sources of astaxanthin.^15,16^

Thraustochytrids, a group of osmoheterotrophic straminipilan marine protists in the Class Labyrinthula (Kingdom Chromista), are predominantly found in estuarine and mangrove ecosystems, where they serve vital roles as detritivores, bacterivores, and parasites.^17^ Notable genera such as *Schizochytrium*, *Thraustochytrium*, and *Ulkenia* are recognized for their remarkable ability to produce substantial quantities of DHA and various secondary metabolites.^11^ These organisms are characterized by high biomass yields and rapid growth rates, with DHA typically constituting an impressive 45–47% of their total fatty acid profile.^18^ Moreover, some strains of thraustochytrids also proficiently biosynthesize carotenoids, including astaxanthin, zeaxanthin, canthaxanthin, β-carotene, echinenone, and phoenicoxanthin.^19,20^ These exceptional features firmly establish thraustochytrids as promising candidates for commercial biotechnological applications, particularly in aquaculture.

However, beyond the varied biochemical profiles, the considerable diversity among thraustochytrid strains with inconsistent morphological features, such as biflagellate gametes and rhizoid-like hyphae,^21^ and their ambiguous placement outside true fungi (the Phycomycetes) or Oomycetes, complicates their taxonomic identification. Consequently, a rapid and reliable method for species-level identification of thraustochytrids remains challenging and is urgently needed. Since the first isolation of *Thraustochytrium proliferum* from the marine alga *Bryopsis plumose* in 1934,^22^ molecular tools such as 18S rRNA sequencing have improved classification. However, species-level identification remains labor-intensive and unreliable for rapid strain screening from environmental samples.^23,24^ Misidentification can result in poor strain selection, ultimately reducing production efficiency and compromising product quality in aquaculture applications.

To address the gap, we explore the use of matrix-assisted laser desorption/ionization time-of-flight mass spectrometry (MALDI-TOF MS), which is widely recognized as one of the most sensitive detection techniques in biological and biochemical research.^25^ In particular, MALDI-TOF mass spectrometry enables the detection of macromolecules, such as proteins and ribosomal peptides, directly from microbial samples with or without prior extraction.^26^ The resulting macromolecular signatures can be used to generate mass spectral profiles, which are then analyzed and clustered into a dendrogram using Biotyper software to facilitate microbial classification.^27,28^ Due to its high sensitivity, rapid data acquisition, and ease of operation, the MALDI-TOF Biotyper system is considered a powerful alternative to conventional methods for microbial species identification.^29–31^ Although it has been widely adopted in clinical microbiology, its application in the taxonomy of marine protists, such as thraustochytrids, remains limited. In this study, we report the establishment of a MALDI-TOF MS-based Biotyper database for Formosan thraustochytrid strains and evaluate its effectiveness in species discrimination in comparison with 18S rRNA phylogenetic analysis.

Incorporating DHA and astaxanthin derived from thraustochytrids into aquaculture feed has shown notable benefits in several key species. Earlier studies have demonstrated that DHA from *Schizochytrium* spp. enhances growth in *Litopenaeus vannamei*,^1^ while astaxanthin in *Aurantiochytrium* sp. improves survival, growth, and immune responses in *Penaeus monodon*.^32^ Our current research reveals that various strains of Formosan thraustochytrids produce high levels of both DHA and astaxanthin. Building on this, we explored the potential of selected thraustochytrid strains as nutritional supplements in shrimp farming, focusing on two strains, one rich in DHA and the other in astaxanthin, as alternative feed additives. This strategy expands the use of marine microbial resources, supporting sustainable and functional shrimp aquaculture.

## Materials and Methods

### General Experimental Procedures

Matrix-assisted laser desorption/ionization time-of-flight mass spectrometry in this study was performed using a Microflex instrument (Bruker Daltonics, Bremen, Germany) equipped with the Bruker Biotyper platform for spectral acquisition and analysis of thraustochytrid proteomic profiles. High-performance liquid chromatography (HPLC) was carried out on a Hitachi L-2310 series pump (Hitachi, Tokyo, Japan) coupled with an L-2420 UV–vis detector (Hitachi, Tokyo, Japan). A reversed-phase C18 column (Discovery HS C18, 5 μm, 250 × 10 mm; Supelco, Bellefonte, PA, USA) was used for chromatographic separation. A Bruker amaZon SL ion trap mass spectrometer (Bruker Daltonics, Bremen, Germany) equipped with an electrospray ionization (ESI) source was employed to acquire mass data for structural confirmation of DHA and astaxanthin.

### Chemicals and Standards

HPLC-grade methanol, acetonitrile, and dichloromethane were obtained from Sigma-Aldrich (St. Louis, USA). The standards, DHA (CAS Number 6217-54-5) and astaxanthin (CAS Number 472-61-7), were purchased from Sigma-Aldrich. For MALDI-TOF MS analysis, the matrices α-cyano-4-hydroxycinnamic acid (CHCA, CAS number 28166-41-8) and sinapic acid (SA, CAS Number 530-59-6), as well as trifluoroacetic acid (TFA, CAS Number 76-05-1), were also obtained from Sigma-Aldrich (St. Louis, USA). All reagents were of analytical-grade or higher purity.

### The Collection and Purification of Thraustochytrids from Formosan Mangrove Areas

Sediment samples were collected from the mangrove areas of Aogu Wetland, Qigu, SiCao, Kaohsiung, and DaPeng Bay in Taiwan and Dongsha Island (see Figure S1 in Supporting Information). The samples were filtered through a 100 mm filter net, followed by a 10 mm filter net. 200 μL filtrate was spread on an AKS medium plate comprising 10 g/L glucose, 0.5 g/L yeast extract, 1 g/L tryptone, 15 g/L agar, 0.1 g/L ampicillin, 0.1 g/L kanamycin, 0.1 g/L streptomycin, and 15 g/L natural sea salt (Red Sea Salt, Red Sea, Houston, TX, USA) and incubated at 28 °C for 14 days. Each thraustochytrid colony was confirmed based on morphology under a microscope and then subcultured onto a new plate for an additional 14 days. Finally, all the collected thraustochytrid strains were stored at −80 °C by frozen preservation (mixed with 200 μL sterile glycerol and 800 μL culture algae solution). In the present study, 19 strains of Formosan thraustochytrids were isolated from the mangrove water and sediments at Aogu Wetland, Qigu, SiCao, Kaohsiung, and DaPeng Bay in southern Taiwan and Dongsha Island (see Figure S1 in Supporting Information), on selective AKS antibiotic medium by repeated three times for concordant confirmation, while 14 strains of thraustochytrids were donated by Prof. Wen-Ming Chen and Prof. Jo-Shu Chang (see Supporting Table S1)

### 18S rRNA Sequencing of Formosan Thraustochytrids (DNA Extraction and Sequencing)

The total genomic DNA of each strain was extracted using the Genomic DNA Mini Kit (Plant) (Geneaid, New Taipei City, Taiwan) following the manufacturer’s instructions. Partial sequences of the nuclear gene 18S rRNA were amplified by polymerase chain reaction (PCR) using two primer sets: SR01 and SR05, 586 and 1286.^33,34^ The PCR solution contained 40 ng of template DNA, 5 μL Taq DNA Polymerase Master Mix (1.5 mM MgCl_2_; Ampliqon, Odense, Denmark), 1 μM of each primer, and ddH_2_O for a final volume of 10 μL. The reaction was performed under the following conditions: 90 s at 95 °C for initial denaturation, followed by 35 cycles of 30 s at 95 °C, 15 s at 53–65 °C, and 50 s at 72 °C with a final extension of 5 min at 72 °C. The amplified fragments were then subcloned into a pET21 vector and sequenced on an ABI-Prism automated DNA sequencer (by Genomics, Taipei, Taiwan). The sequences of each sample were assembled and edited in Geneious 8.1.8 (https://www.geneious.com). Sequences were aligned with CLUSTAL X^35^ using default settings, adjusted by eye in Geneious 8.1.8, and then trimmed to remove poorly aligned regions and gaps at sequence termini.^36^ The best substitution model was selected using a corrected Akaike Information Criterion (AICc) and applied for phylogenetic reconstruction via the Maximum-Likelihood (ML) method implemented in MEGA X.^37^ Node support was estimated using 5,000 bootstrap replicates.^38^ Species names in the phylogenetic tree were assigned based on BLAST analysis against the GenBank database, with each strain named according to the reference species showing the highest percent identity value (greater than 98%).^36^

### Culture of Thraustochytrids

Frozen preservation stocks containing thraustochytrid strains were defrosted, and 150 μL of the stock was transferred with a micropipette into a tube containing 3 mL of medium broth. The cultures were inoculated at 150 rpm and 28 °C for 72 h. Among them, only 33 strains of thraustochytrids, collected from the mangrove areas of Taiwan and Dongsha Island, could be cultivated. The culturable strains were further trans-cultivated on the solid modified GYPA medium (comprised of 10 g/L glucose, 0.5 g/L yeast extract, 1 g/L tryptone, 15 g/L agar, and 15 g/L natural sea salt) at 28 °C for 7 days. Each colony of thraustochytrid strains was confirmed and identified by microscopic morphology, then subcultured on a new plate for an additional 7 days. The microscopic images and colony morphologies of representative thraustochytrid strains are shown in Figure S2 in the Supporting Information. Further, each thraustochytrid strain was trans-cultivated into a 25 mL tube containing 3 mL sterile GYPA broth, incubated at 150 rpm and 25 °C for 72 h for the MALDI-TOF MS spectral measurement.

### Protocol Selection to Prepare Thraustochytrid Samples

The sample preparation protocol was optimized using *Aurantiochytrium* sp. YA7.1 as a representative strain. Four extraction methods were evaluated for matrix selection (Figure S3 in Supporting Information): Method 1, acetonitrile extraction with sinapic acid (SA) overlay; Method 2, direct SA extraction; Method 3, formic acid extraction with SA overlay; and Method 4, trifluoroacetic acid extraction with α-cyano-4-hydroxycinnamic acid (CHCA) overlay. Method 2 yielded the best performance in terms of signal-to-noise ratio, reproducible peak count, and spectral consistency (Figure S3 in Supporting Information). To optimize cultivation, a six-day time-course analysis revealed that day 3 provided optimal conditions for protein profiling (Figure S4 in Supporting Information). Based on these results, Method 2, combined with a three-day culture period, was selected as the standard protocol for MALDI-TOF MS analysis of all 33 thraustochytrid strains.

### Sample Preparation for MALDI-TOF MS Measuring

The thraustochytrid cells were harvested after 3 days of cultivation by transferring 500 μL of culture suspension into a 2 mL Eppendorf tube, followed by centrifugation at 12,000 rpm for 2 min at room temperature. After carefully discarding the supernatant, the cell pellet was retained. To the pellet, 40 μL of freshly prepared SA matrix solution was added, along with 6–7 stainless steel beads (2 mm diameter). The mixture was vigorously vortexed for 5 min to promote mechanical cell disruption and protein extraction, then centrifuged at 10,000 rpm for 2 min. The resulting supernatant, containing extracted proteins and matrix, was collected for MALDI-TOF MS analysis. The SA matrix solution was freshly prepared at a concentration of 20 mg/mL in 50% acetonitrile and 0.1% trifluoroacetic acid (TFA).

### MALDI-TOF MS Analysis of Thraustochytrids

The aforementioned supernatant with the matrix solution (1 μL) was dropped onto an MSP 96 target polished steel plate (Bruker Daltonics, Bremen, Germany) and allowed to dry at room temperature. For each strain, five replicate target positions were prepared to ensure measurement reproducibility. Mass spectra were acquired using an Autoflex Speed MALDI-TOF/TOF mass spectrometer (Bruker Daltonics, Bremen, Germany) operated with flexControl software version 3.0 (Bruker Daltonics). Spectra were recorded in linear positive-ion mode over the *m*/z range of 2,000 to 20,000 (Detector Gain 8.1 × 2,819 V) and a laser frequency of 500 Hz. For each strain, four replicate target spots were prepared. For each sample, 2,000 laser shots were applied in four sets of 500 shots at different positions, with the four subacquisitions automatically averaged to generate one spectrum per acquisition. Five spectra were collected from each of the four replicate targets, yielding a total of 20 technical replicate mass spectra per strain (4 spots × 5 acquisitions = 20 spectra). This multilevel replication accounts for within-spot and between-spot variability. The use of multiple technical replicate spots is standard practice in MALDI-TOF MS to ensure measurement reproducibility and minimize spot-to-spot variability.^39,40^ The instrument was calibrated using Protein Calibration Standard I (Bruker Daltonics). *Aurantiochytrium* sp. AP45 was analyzed in parallel with the samples as a positive control, consistently yielding identification scores higher than 2.3 when compared against the AP45 Main Spectrum Profile in the MALDI Biotyper database, thereby validating system performance throughout the study period.

### Raw Mass Spectra Processing

Raw mass spectra were processed using MALDI Biotyper software version 3.1 (Bruker Daltonics) according to the Biotyper Preprocessing Standard Method. Preprocessing parameters included: smoothing method: Savitzky–Golay filter; baseline subtraction method: multipolygon; normalization method: maximum normalization. The mass range was restricted to *m*/z 2,000–20,000 (the default range for protein profiling). Peak detection was performed using the spectral classification method with a signal-to-baseline noise ratio threshold of 3, identifying up to 100 peaks per individual spectrum.

### Main Spectrum Profile (MSP) Creation and Database Construction

For each strain, the 20 processed technical replicate spectra were consolidated by MALDI Biotyper software to generate a single MSP per strain, following the established Biotyper MSP creation workflow. MSPs are Bruker Biotyper-specific reference spectra that represent the consensus proteomic fingerprint of a microorganism. The automated MSP creation algorithm performs peak alignment across replicate measurements, applies frequency-based peak filtering (minimum peak frequency threshold: 60%, retaining peaks appearing in at least 12 of 20 replicates), and calculates average peak intensities to generate a high-quality representative spectrum with minimized noise and artifact signals.^41^ A total of 33 strain-specific MSPs (one MSP per strain) were created and used for constructing a custom thraustochytrid reference database and subsequent taxonomic analysis. According to established Bruker Biotyper scoring criteria, MSP similarity scores ≥2.0 indicate reliable identification at genus and probable species levels, scores 1.7–1.999 indicate probable genus identification, while scores <1.7 are considered unreliable.^42,43^

### Hierarchical Clustering Analysis

Given the absence of a comprehensive MALDI-TOF MS reference database for thraustochytrids, we adopted an unsupervised clustering approach based on proteomic profiles. Hierarchical clustering dendrograms were constructed using the MALDI Biotyper MSP Dendrogram Creation Standard Method (Bruker Daltonics) with correlation coefficient as the distance metric and average linkage (UPGMA) for clustering. Proteomic dissimilarities were assessed using ribosomal protein mass fingerprints in the *m*/z range of 2,000–20,000, with higher values indicating greater divergence between strains. A total of 33 MSPs were clustered based on proteomic similarities, independent of any prior taxonomic information. The resulting dendrogram was interpreted as a proxy for taxonomic relatedness and subsequently validated by comparison with phylogenetic relationships inferred from 18S rRNA gene sequences.

### HPLC Quantitative Analysis of Docosahexaenoic Acid (DHA) and Astaxanthin from Thraustochytrids

Thraustochytrid strains were individually cultivated in 125 mL flasks containing 10 mL of autoclaved modified GYPA medium, incubated at 150 rpm and 28 °C for 3 days. Cultures were harvested by centrifugation at 4,000 rpm, the supernatant was removed, and cell pellets were freeze-dried for 24 h. Dried cells were extracted with acetone using bead vortexing for 5 min to obtain organic layers containing DHA and astaxanthin, and the extract was filtered through a 0.22 μm syringe filter prior to HPLC analysis.

Quantitative HPLC analysis was performed using a Supelco Discovery HS C18 column (5 μm, 250 × 4.6 mm) at a flow rate of 1.0 mL/min. Due to the distinct chemical properties of the target compounds, separate analytical runs were performed for DHA and astaxanthin. DHA was analyzed using isocratic elution with methanol/ddH_2_O + 0.1% TFA (95:5, v/v) and detected at λ = 210 nm. Astaxanthin was analyzed using gradient elution with a mixture of acetonitrile, methanol, dichloromethane, and ddH_2_O containing 0.1% TFA (gradient program detailed in Supplementary Table S2), with detection at 480 nm. External calibration curves were established using standard solutions of DHA (100 ppm) and astaxanthin (1 ppm), injected at varying volumes (1, 2, 5, 10, 20 μL). Data were acquired and processed using D-2000 Elite software, and compound concentrations were determined by HPLC calibration.

### Using Isolated Thraustochytrids as Nutritional Supplements for White Shrimp Farming

According to analytical results of high-value compounds in thraustochytrids, two strains, YA7.1 (*Aurantiochytrium sp.*) and W1.2 (*Schizochytrium* sp.), rich in astaxanthin and DHA, respectively, were selected to ferment for mass production.

Fermentation condition: the bioreactors (FIRSTEK FB-6S, FIRSTEK AIR PUMP, FIRSTEK B401H cooler) contained 10 L GYP broth media, maintained at 150 rpm agitation, 28 °C temperature, and 1.5 vvm air input. Additionally, using a foam detector, when the algae and culture medium are continuously stirred and aerated, the foam produced reaches the detection limit, a defoamer is dripped to eliminate the foam and prevent the culture medium from splashing out of the tank.

### Whiteleg Shrimp Feeding Test

Postlarval whiteleg shrimps (*Litopenaeus vannamei*) with an initial length of about 0.8 ± 0.1 cm were purchased from Yiyuan Aquaculture Company (Kaohsiung, Taiwan). Commercial feed was sourced from a supplier in Pingtung. The feeding experiment was conducted in two stages. Based on prior studies demonstrating that a 3% inclusion level of *Aurantiochytrium* sp. meal effectively enhances disease resistance and survival rates in Pacific white shrimp without negatively impacting growth performance,^44^ we adopted the same 3% supplementation level in this study to evaluate the nutritional and functional benefits of selected thraustochytrid strains. In the first stage, approximately 100 postlarval *L. vannamei* were stocked per tank. Tanks were maintained at a controlled temperature of 26 ± 1 °C and salinity of 31 ± 2 practical salinity units (PSU) throughout the experimental period. Postlarvae were randomly assigned to one of three dietary treatments, each replicated three times: (1) control diet (regular commercial feed), (2) *Schizochytrium*-supplemented diet (regular feed +3% *Schizochytrium* sp. W1.2 powder), and (3) *Aurantiochytrium*-supplemented diet (regular feed +3% *Aurantiochytrium* sp. YA7.1 powder). Shrimp length and survival were recorded weekly. In the second stage, a total of 2700 adult shrimp (initial body weight: 7.91 ± 0.17 g) were individually weighed and divided into three groups consisting of 900 shrimp each. Each group was placed into a separate pond with a surface area of 25 m^2^ and a water volume of approximately 37.5 m^3^ (maximum depth: 1.5 m). Shrimp were weighed weekly at four sampling points per pond, and total numbers were recorded at harvest during the final week.

### Performance Parameters

The experimental postlarval stage period lasted 36 days, and the adult stage period lasted 56 days. Performance parameters of shrimp were calculated as

1 $$Survival\;Rate\;\left(SR,\%\right)=\left(\frac{Final\;number\;of\;shrimp}{Initial\;number\;of\;shrimp}\right)\times 100$$

2 $$Specific\;Growth\;Rate\;(\mathrm{BW})\;\left(SGR,\%\;perday\right)=\left(\frac{ln\left(W_{t}\right)-\ln\left(W_{i}\right)}{T}\right)\times 100$$

3 $$Specific\;Growth\;Rate\;(BL)\;\left(SGR,\%\;perday\right)=\left(\frac{ln\left(L_{t}\right)-\ln\left(L_{i}\right)}{T}\right)\times 100$$

4 $$Food\;Conversion\;Ratio\;\left(FCR,\%\right)=\left(\frac{Total\;Feed\;Intake\;\left(g\right)}{Total\;Weight\;Gain\;\left(g\right)}\right)\times 100$$

5 $$Food\;Conversion\;Efficiency\;\left(FCE,\%\right)=\left(\frac{Total\;Weight\;Gain\;\left(g\right)}{Total\;Feed\;Intake\;\left(g\right)}\right)\times 100$$

Where *ln*: natural logarithm, *BW*: body weight of the shrimp, *W_i_*: average weight initial (g), *W_t_*: average weight after feeding (g), *BL*: body length of the shrimp, *L_i_*: average length initial (cm), *L_t_*: average length after feeding (cm).

### Statistical Analysis

Data visualization and preliminary analysis of DHA, astaxanthin, and biomass parameters were performed using OriginLab (OriginLab Corporation, Northampton, MA, USA). Multivariate analysis for strain grouping employed a two-step approach combining PCA and K-means clustering. PCA was conducted on six production variables: DHA content (mg/g), DHA yield (mg/L), astaxanthin content (μg/g), astaxanthin yield (μg/L), and biomass from both optimization batches. Variables were standardized before analysis. K-means clustering (k = 4) was performed on the first two principal components (PC1-PC2) to identify production groups. Analyses were conducted using RStudio (R Core Team, Vienna, Austria).

For the aquaculture trial, IBM SPSS Statistics 28.0 (SPSS Inc., Chicago, IL, USA) was utilized for statistical analysis of *L. vannamei* performance parameters. All data were subjected to Levene’s and Shapiro–Wilk tests to assess homoscedasticity and normality, respectively. The survival rate (SR), growth performance, and food efficiency of *L. vannamei* were evaluated using repeated measures ANOVA (RM-ANOVA). The general linear model procedure was employed to assess each response variable and determine whether significant differences existed among food additive treatments as factors. When significant differences were found in the interaction terms, post hoc comparisons were performed using the least significant difference (LSD) test. All statistical tests were performed using a significance level of 5% (α = 0.05). The categorical distribution of proportion size was analyzed using the Chi-square test.

### Ethical Statement

Whiteleg shrimp (*Litopenaeus vannamei*) used in this study are invertebrate animals and are not classified as experimental animals requiring ethical approval under the regulations of Taiwan. Therefore, approval from an Institutional Animal Care and Use Committee (IACUC) was not required. All experimental procedures followed standard aquaculture practices and institutional guidelines to minimize stress and ensure humane handling of the animals. Shrimp were maintained under species-appropriate environmental conditions (water temperature 26 ± 1 °C, salinity 31 ± 2 PSU) throughout the study to ensure optimal animal welfare.

## Results and Discussion

A total of 33 strains of Formosan thraustochytrids were isolated from Taiwan mangrove environments (see Figure S1 in the Supporting Information). These strains were cultured in selective AKS antibiotic medium and subjected to 18S rRNA gene sequencing for phylogenetic classification and mass dendrogram Biotyper analysis.

### The Phylogenetic Relationships of Formosan Thraustochytrid Strains

The 18S rRNA phylogenetic tree constructed from all 33 Formosan thraustochytrid strains revealed three main clades with strong bootstrap support, along with a miscellaneous group of unassigned strains (see Figure 1). Clade 1 was the largest group, comprising 19 strains affiliated with the genera *Aurantiochytrium* and *Thraustochytrium*. Clade 2 contained three strains of *Aurantiochytrium*, while Clade 3 included four strains of *Schizochytrium*. The remaining seven strains could not be confidently assigned to any of the three main clades and were therefore classified as the miscellaneous group. Strains within each clade exhibited high sequence similarity in their 18S rRNA genes, supporting their phylogenetic grouping.

**Figure 1:**
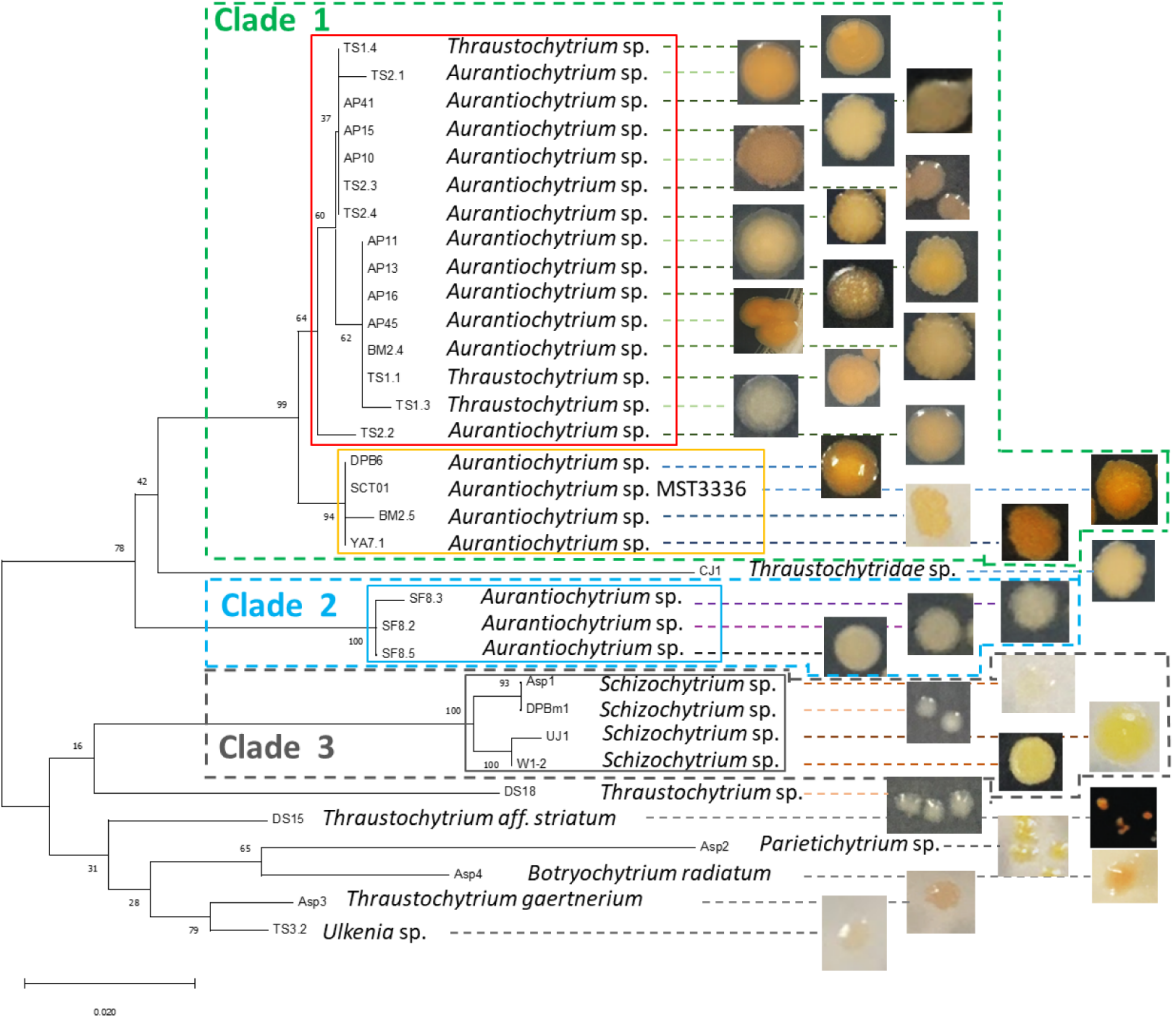
The maximum likelihood phylogenetic tree of 33 Formosan thraustochytrid strains, showing three main clades and a miscellaneous group. Clade 1 (green dashed line): composed of *Aurantiochytrium* and *Thraustochytrium* strains; Clade 2 (blue dashed line): composed of *Aurantiochytrium* strains; Clade 3 (gray dashed line): dominated by *Schizochytrium* species. Bootstrap support values (>50%) are shown at the nodes. Scale bar: 0.020 substitutions per site. Color boxes indicated correspondence with MALDI-TOF groups (Figure 2): Clade 1 splits into Group 2 (yellow solid line: high-astaxanthin producers) and Group 4 (red solid line: high-biomass producers); Clade 2 corresponds to Group 1 (blue solid). Clade 3 corresponds to Group 3 (gray solid line: high DHA producers). Miscellaneous strains correspond to a distinct cluster (purple).

Microscopic observation revealed conserved cellular morphological features among isolates, including spherical-ovoid cells (10–30 μm) and irregular colonies with smooth margins (Figure S2 in Supporting Information), providing insufficient resolution for taxonomic discrimination. However, colony pigmentation exhibited clade-specific patterns that correlated with biochemical traits: Clade 1 strains displayed orange-yellow pigmentation, Clade 2 ones were brownish-orange, Clade 3 (*Schizochytrium*) formed pale colonies, and miscellaneous strains displayed variable coloration. Notably, the orange-yellow colonies of Clade 1 corresponded with elevated astaxanthin production (Figures 3 and 4). Highlighting the potential utility of pigmentation as a phenotypic marker for biochemical capacity. Clade 2 displayed darker brownish-orange pigmentation, and both Clades 1 and 2 suggested carotenoid production (e.g., astaxanthin, phoenicoxanthin, canthaxanthin, or beta-carotene).^45^ While Clade 3, represented by *Schizochytrium* spp., was particularly distinct from the other clades both genetically and morphologically, these strains formed characteristically pale yellow colonies, implying low pigment content but high PUFAs production, particularly DHA.^45^ The high DHA contents of these strains were confirmed by further quantitative analysis. In contrast, the miscellaneous group displayed variable pigmentation.

Despite exhibiting identical colony pigmentation and conserved cellular morphology (Figure S2 in Supporting Information), the Clade 1 strains *Aurantiochytrium* spp. AP11 and YA7.1 exhibited markedly different biochemical profiles. Strain AP11 (Group 4) demonstrated high biomass production with balanced biochemical traits, whereas YA7.1 (Group 2) specialized in astaxanthin accumulation (Figure 4). This intraclade functional divergence underscores the limitations of morphological and phylogenetic criteria alone in predicting biotechnologically relevant phenotypes. While MALDI-TOF MS-based proteomic fingerprinting provides rapid, high-resolution discrimination at the strain level, surpassing traditional morphology or sequence-based methods, direct biochemical profiling remains indispensable for identifying specific biosynthetic capabilities. Although colony pigmentation trends were partially congruent with phylogenetic clades (notably in Clades 2 and 3), the pronounced biochemical heterogeneity within the morphologically uniform Clade 1 reveals underlying diversity in biosynthetic pathway regulation among closely related strains. These findings reinforce the value of integrated classification approaches combining morphological, phylogenetic, proteomic, and metabolic data to inform the selection of high-performing strains for functional and industrial applications.

### Biotyper Identification of Formosan Thraustochytrid Strains

To facilitate rapid and accurate strain identification, we established a MALDI-TOF MS-based Biotyper database encompassing 33 Formosan thraustochytrid strains. Protein profiling was optimized using SA as the matrix in a direct extraction protocol (Figure S3 in Supporting Information). The biological relevance of resulting proteomic fingerprints was validated by strong concordance between MALDI-TOF MS clustering and independent 18S rRNA phylogenetic analysis (Figures 1–2). It is well-known that thraustochytrid strains exhibit diverse optimal growth conditions, which can significantly influence their proteomic profiles, particularly in ribosomal protein expression, depending on variables such as nutrient availability, incubation time, and temperature. Therefore, all strains were cultured under standard conditions in modified GYPA medium at 28 °C. To determine the optimal incubation time for protein-based profiling, we conducted a preliminary assessment by culturing a selected strain, *Aurantiochytrium* sp. YA7.1, for 1 to 6 days. Samples were prepared for MALDI-TOF MS analysis using bead vortexing. The chosen strain exhibited optimal spectral quality with the strongest and most reproducible mass spectra profiles on day 3 (Figure S4 in Supporting Information), which was subsequently used as the standard cultivation time point for generating MSPs for all strains.

**Figure 2:**
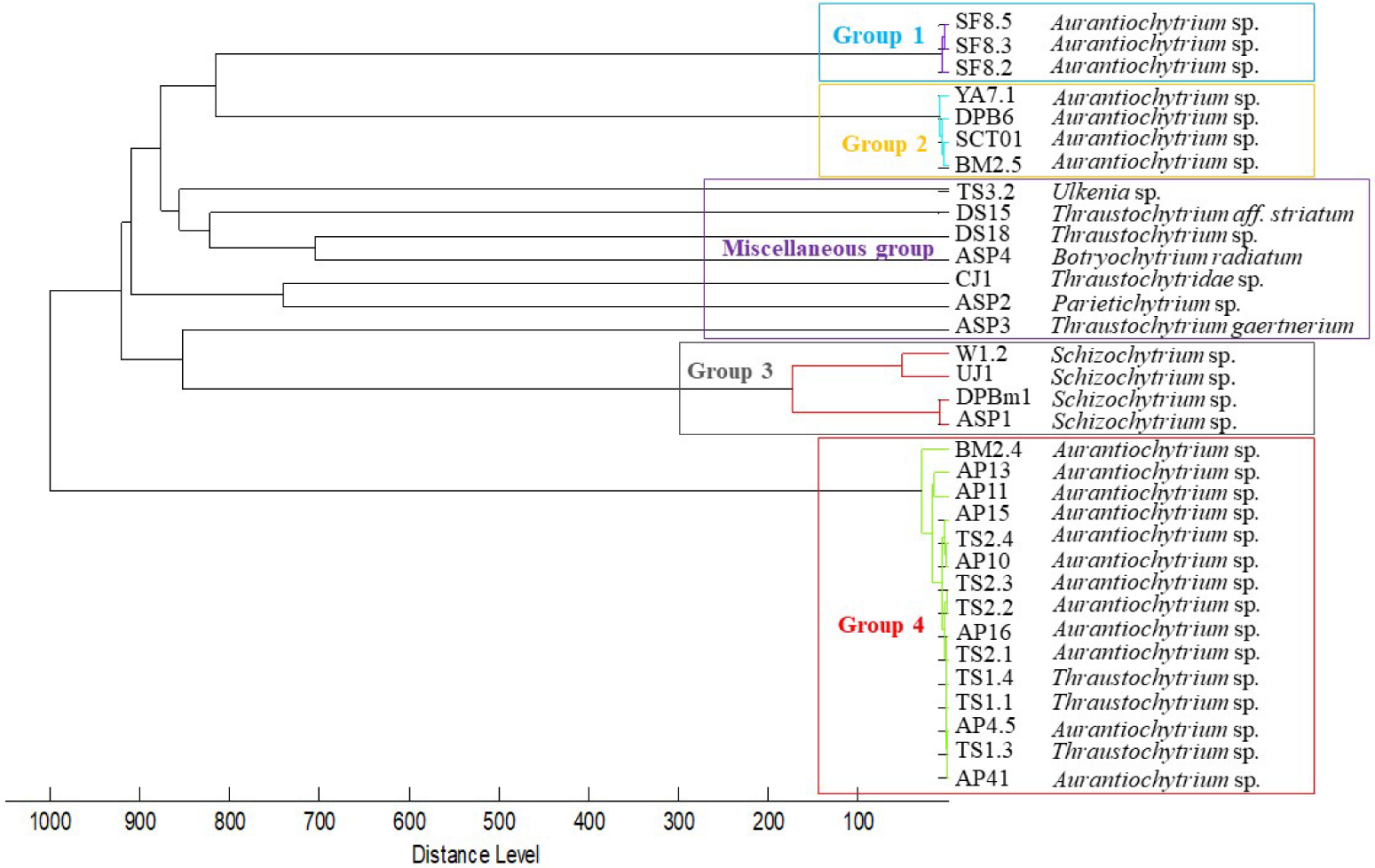
Hierarchical clustering of 33 Formosan thraustochytrid strains based on MALDI-TOF MS proteomic profiles, performed using the Biotyper MSP Dendrogram Creation Standard Method (UPGMA, correlation coefficient distance) on MSPs derived from the *m*/*z* range of 2,000–20,000. The *x*-axis indicates proteomic dissimilarity, calculated from the correlation coefficient, with higher values reflecting greater divergence in protein fingerprints. The analysis resolved four main groups and one miscellaneous cluster. Colored boxes correspond to phylogenetic clades in Figure 1. Group 1 (blue solid) matches Clade 2. Groups 2 (yellow solid) and 4 (red solid) are subsets of Clade 1. Group 3 (gray solid) corresponds to Clade 3. Miscellaneous (purple) are taxonomically divergent strains. Proteomic clustering captures both phylogenetic relationships and diversity in protein expression profiles among strains.

According to Biotyper scoring criteria, an MSP score ≥2.0 indicates reliable identification at both species and genus levels, while scores <1.7 are considered unreliable and categorized as “miscellaneous”. Unsupervised hierarchical clustering of the 33 thraustochytrid MSPs classified into four distinct groups and one miscellaneous (see Figure 2). Group 1 included three isolates that corresponded with Clade 2 from the 18S rRNA phylogenetic analysis. Group 3 contained four isolates matching Clade 3. In contrast, the 19 strains classified in Clade 1 based on 18S rRNA data yielded inconsistent Biotyper scores, ranging from 1.7 to 2.0, indicating only genus-level identification. These strains were further subdivided into two distinct groups, Groups 2 and 4, suggesting proteomic divergence within the clade. In contrast, the seven isolates that did not belong to the genera *Aurantiochytrium* or *Schizochytrium* also failed to meet the Biotyper reliable identification threshold (MSP similarity scores <1.7, indicating unreliable identification according to established Biotyper scoring criteria, and were classified as the Miscellaneous group.

The phylogenetic tree of Formosan thraustochytrids, constructed using 18S rRNA gene sequences, illustrates the distinct relationships among 33 strains, which were grouped into three well-supported clades and one miscellaneous group. In parallel, the MSP dendrogram generated from MALDI-TOF MS Biotyper analysis also revealed distinct clustering patterns, dividing the same 33 strains into five groups. A side-by-side comparison of the MSP dendrogram and the 18S rRNA-based phylogenetic tree (see Figures 1 and 2, Table S3) was performed to clarify the similarities and differences between proteotypic and genotypic classification of Formosan thraustochytrid strains. Clade 1 from the genotypic phylogeny was split into two groups, Groups 2 and 4, in the MSP dendrogram. In contrast, Clades 2 and 3 corresponded directly to Groups 1 and 3, respectively. Genotype-based phylogeny is widely recognized as a robust method for elucidating microbial taxonomic relationships.^46^ However, the Biotyper system offers a complementary perspective by bridging the gap between genotypic information and macromolecular phenotypes, particularly protein and peptide profiles captured by MALDI-TOF MS.^47^ Based on this analysis, this division suggests potential biochemical or proteotypic differentiation within Clade 1, with Group 2 and Group 4 representing distinct subgroups. However, further genetic or taxonomic evidence is required to determine whether these reflect subgeneric variation or species-level distinctions. The proteomic fingerprints generated by MALDI-TOF MS enhance strain-level resolution by capturing phenotypic differences that may not be apparent through DNA sequencing alone. This MALDI-TOF MS-based approach can provide valuable information for taxonomic revision within thraustochytrids, in addition to 18S rRNA and morphological data.^48^

Despite its advantages, MALDI-TOF MS analysis has several limitations. These include its reliance on reference databases,^49^ relatively high detection thresholds (ranging from 10^2^ to 10^4^ cells),^43^ and reduced discriminatory power when protein profiles are highly similar among closely related species.^50,51^ These challenges are particularly relevant for environmental isolates, such as thraustochytrids, which are underrepresented in most commercial databases. Alternative microbial identification methods include metagenomic next-generation sequencing (mNGS) and multilocus sequence typing (MLST). mNGS enables comprehensive, culture-independent detection of both known and novel organisms in complex microbial communities. However, it is costly, bioinformatically intensive, and often hindered by lengthy processing times and host DNA contamination.^52,53^ MLST, regarded as the gold standard for microbial genotyping, offers excellent reproducibility and taxonomic resolution through the analysis of multiple housekeeping genes. Nevertheless, it is labor-intensive, expensive, and impractical for high-throughput or routine applications.^54^ Compared to these alternatives, the Biotyper system offers a more practical and cost-effective option, especially when integrated with conventional 18S rRNA sequencing. This combined genotypic-phenotypic approach enhanced classification confidence and provides a more comprehensive framework for the taxonomic characterization of thraustochytrids.

### Analysis of DHA Production in Formosan Thraustochytrids

We further analyzed the ability of Formosan thraustochytrid strains to produce high-value compounds, particularly DHA and astaxanthin. Of the 33 strains, only 26 from Groups 1 to 4 were included in this analysis. Seven strains from the miscellaneous group (ASP2, ASP3, ASP4, TS3.2, CJ1, DS15, and DS18) were excluded because they exhibited insufficient growth following amplification. In addition to biochemical composition, biomass production was evaluated for each strain, as it is a key determinant of commercial feasibility. Biomass yield is a critical parameter,^45^ and the variation in growth rates among thraustochytrid strains during fermentation can significantly influence DHA accumulation.^55,56^ In our study, notable differences in biomass production were observed among the Formosan strains, even under identical fermentation conditions (see Figure 3). DHA content was measured daily over a five-day cultivation period, with each measurement performed in triplicate. The results reported represent the average of these triplicate measurements. Detailed daily DHA production data for each strain (*n* = 3) are illustrated in a bar chart (see Figure S5 in Supporting Information).

**Figure 3:**
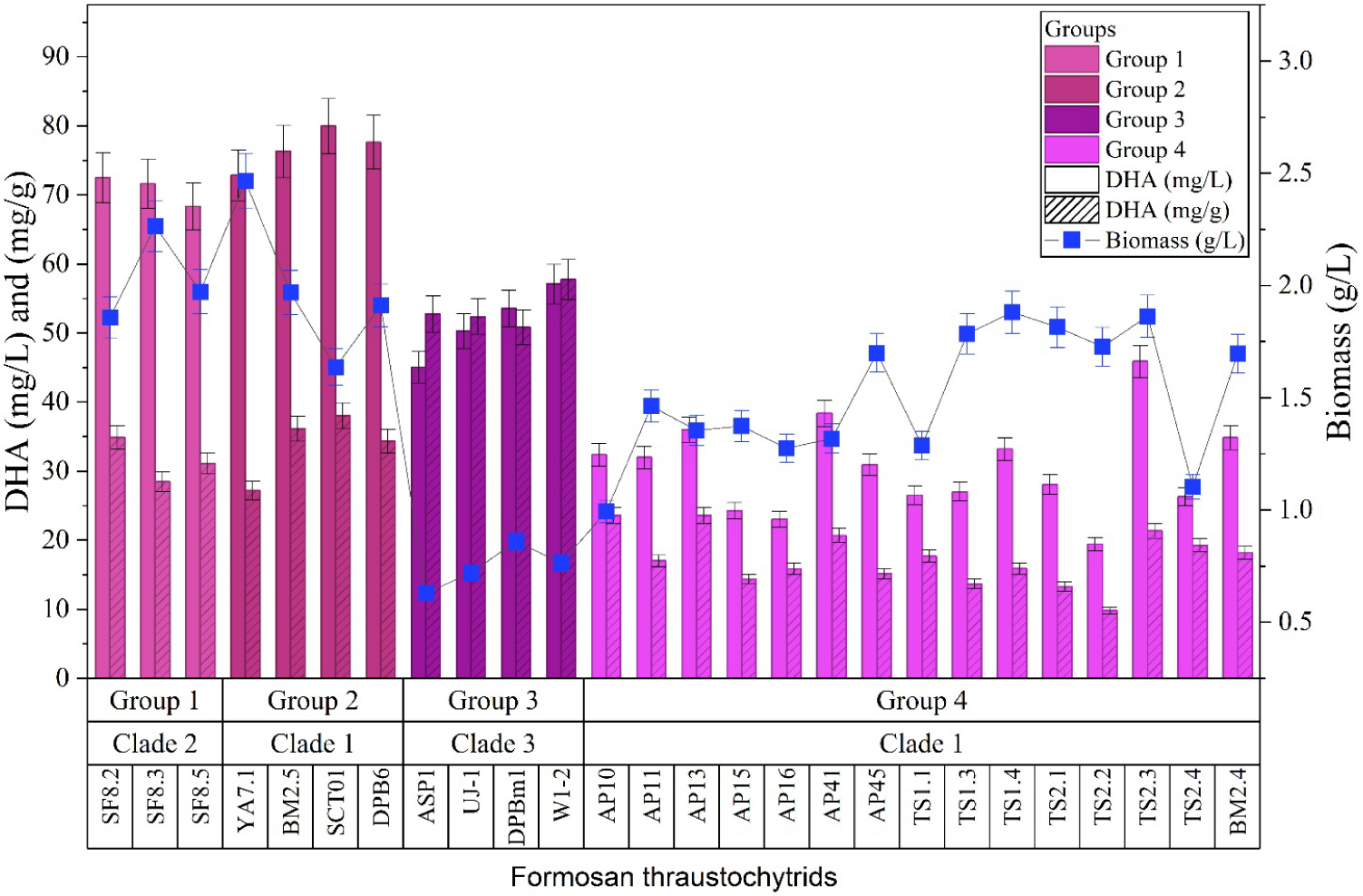
DHA content (mg/g), total DHA yield (mg/L), and biomass yield (g/L) at the time of maximum DHA production (typically day 2–3; see Figure S5 in Supporting Information for time-course data). Strains are arranged according to proteomic classification. Error bars: SD (*n* = 3). *Schizochytrium* strains (Group 3) showed the highest DHA per unit biomass, whereas total yield varied depending on biomass accumulation.

Among the analyzed strains, all *Schizochytrium* species in Group 3 exhibited the highest DHA content, ranging from 51 to 58 mg/g dried cell weight (DCW), with the peak production occurring after 2–3 days of cultivation. However, these strains generally produced lower biomass compared to those in other groups. In contrast, 19 *Aurantiochytrium* strains across Groups 1, 2, and 4 yielded moderate DHA levels, ranging from 9.8 to 38.0 mg/g DCW, with optimal production achieved after a longer fermentation period of 2–3 days. Notably, *Aurantiochytrium* strain SCT01 (Group 2) achieved a relatively high DHA yield of 38.0 mg/g DCW, while strain TS2.2 (Group 4) produced the lowest DHA content at 9.8 mg/g DCW. Literature survey indicated that *Schizochytrium* species generally possess higher DHA productivity,^57,58^ and that their DHA output can be further enhanced through genetic engineering.^59^ These findings suggest that the *Schizochytrium* strains isolated from Taiwan’s mangrove ecosystems hold promising potential for future commercial application in DHA production.^57^

### Analysis of Astaxanthin Production in Formosan Thraustochytrids

Thraustochytrid strains are known to produce commercially valuable carotenoids, with astaxanthin being the predominant pigment.^60^ Accordingly, we quantified astaxanthin production in the Formosan thraustochytrid strains (see Figure 4). Daily monitoring of astaxanthin synthesis revealed that most strains (each tested in triplicate, *n* = 3) achieved peak production after 4–5 days of cultivation (see Figure S6 in Supporting Information). All *Aurantiochytrium* strains from Groups 1, 2, and 4 produced appreciable levels of astaxanthin. Among these, strains in Group 2 had the highest astaxanthin content, while SF-series strains (Group 1) showed comparatively lower levels compared to other *Aurantiochytrium* species. Notably, *Aurantiochytrium* sp. strain YA7.1 achieved the highest astaxanthin concentration, reaching 90.70 μg/g DCW. These findings align with previous reports highlighting the astaxanthin-producing capability of *Aurantiochytrium* species, particularly *A. limacinum* (formerly classified as *Schizochytrium limacinum*),^61^ In contrast, *Schizochytrium* strains in Group 3 produced minimal astaxanthin (3.87 μg/g DCW) and formed pale yellowish-white colonies when cultured on agar plates (see Figure 1), consistent with their low pigment content.

**Figure 4:**
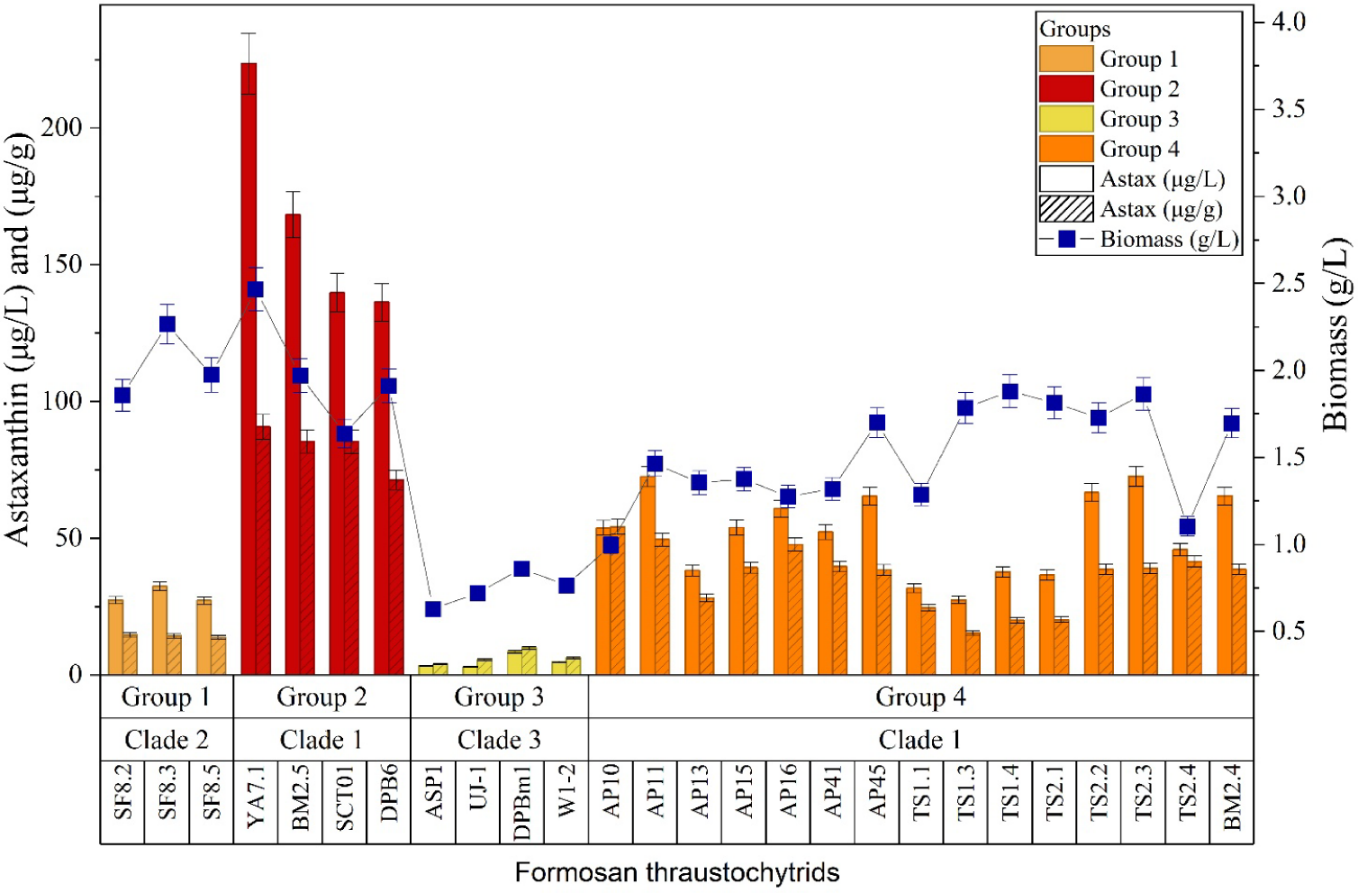
Astaxanthin content (μg/g), total astaxanthin yield (μg/L), and biomass yield (g/L) at the time of maximum astaxanthin production (typically day 4–5; see Figure S6 in Supporting Information for time-course data). Strains are arranged according to proteomic classification. Error bars: SD (*n* = 3). All *Aurantiochytrium* strains produced astaxanthin, with the highest levels per unit biomass in Group 2. *Schizochytrium* strains (Group 3) showed minimal astaxanthin.

### Multivariate Analysis of Biochemical Profiles

Analysis of 26 thraustochytrid strains using principal component analysis (PCA) combined with K-means clustering identified four distinct groups based on DHA and astaxanthin levels, as well as biomass output. The PCA biplot captured 85.8% of the total variance (PC1: 52.3%, PC2: 33.5%), demonstrating effective dimensionality reduction of the biochemical data set. This analysis method successfully differentiated the thraustochytrid strains into meaningful metabolic clusters. Group 2 (*Aurantiochytrium* sp.) showed high astaxanthin levels, while Group 3 (*Schizochytrium* sp.) exhibited elevated DHA content (see Figure S7 in Supporting Information). These biochemical groupings were independently validated by K-means clustering, which identified the same four groups, validating the robustness of the classification strategy (see Figure 5). Importantly, the clustering results from both PCA and K-means aligned with MALDI-TOF MS Biotyper groupings, demonstrating a strong correlation between metabolic profiles and proteomic fingerprinting. Although 18S rRNA-based phylogenetic analysis revealed three major clades, the biochemical clustering further divided Clade 1 into two subgroups with distinct metabolic functions. This indicates significant metabolic diversity within phylogenetically similar species and underscores the importance of integrating biochemical and phenotypic data for precise strain selection in biotechnology.

**Figure 5:**
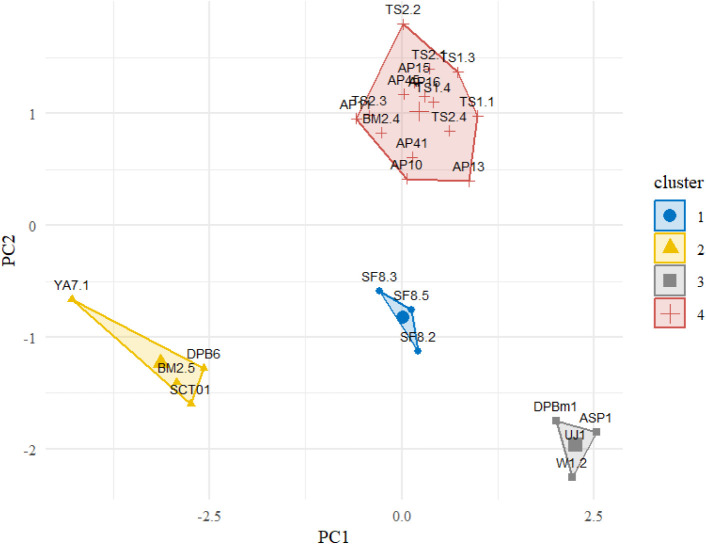
K-means clustering of 26 Formosan thraustochytrid strains applied to PCA-transformed data based on DHA and astaxanthin content, and biomass yield. Four distinct metabolic groups: Group 1 (blue) encompasses moderate biochemical producers, Group 2 (yellow) comprises high-astaxanthin strains, Group 3 (gray) represents high-DHA producers, and Group 4 (red) consists of high-biomass producers with balanced biochemical content.

Based on the PCA result, we selected *Aurantiochytrium* sp. YA7.1 and *Schizochytrium* sp. W1.2 for aquaculture feeding trials, as these two strains from Groups 2 and 3, respectively, exhibited complementary profiles in the production of high-value compounds (DHA and astaxanthin). *Aurantiochytrium* sp. YA7.1 produced both elevated levels of astaxanthin and a moderate amount of DHA, whereas *Schizochytrium* sp. W1.2 demonstrated a high yield of DHA with minimal production of astaxanthin.

### Stage-Specific Benefits of Thraustochytrid Supplementation in Whiteleg Shrimp *Litopenaeus vannamei*

Feeding trials were conducted to assess the effects of *Schizochytrium* sp. W1.2 and *Aurantiochytrium* sp. YA7.1 on the growth and survival of *Litopenaeus vannamei* across two developmental stages: postlarval and adult. The postlarval stage is particularly crucial for shrimp aquaculture, as survival at this stage greatly impacts overall yield. During the 36-day postlarval phase, shrimp were fed with 3% *Schizochytrium* sp. W1.2 powder showed the highest survival rate (57.3%) and superior specific growth rate based on body length (SGR-BL: 4.02%/day), significantly outperforming both the control group (21.3% survival, 3.53%/day SGR-BL) and the group supplemented with *Aurantiochytrium* sp. YA7.1 (34.7% survival, 3.97%/day SGR-BL) (see Table 1). Statistical analysis revealed significant differences among treatments for both survival and growth parameters (F (2.21, 13.27) = 33.395 and F (1.34, 8.04) = 33.395, respectively, *p* < 0.001). The results suggest that the high DHA content in W1.2 plays a key role in supporting neural and visual development during early shrimp growth.^62^

**Table 1:** Parameters Performance of *Litopenaeus vannamei* during the Experimental[Table-fn tbl1fn1]

Parameters	Control	Control +3% powder of *Schizochytrium* sp. W1.2	Control +3% powder of *Aurantiochytrium* YA.7.1
Post-Larval of *L. vannamei*			
Survival Rate (%)	21.33 ± 2.94^c^	57.33 ± 7.69^a^	34.67 ± 1.75^b^
SGR_Body Length (%/day)	3.53 ± 0.12^b^	4.02 ± 0.15^a^	3.97 ± 0.16^a^
Adult of *L. vannamei*			
SGR_Body Weight (%/day)	1.69 ± 0.03^c^	2.15 ± 0.06^a^	1.98 ± 0.03^b^
FCR	2.16 ± 0.35^c^	1.81 ± 0.27^b^	1.64 ± 0.18^a^
FCE (%)	47.29 ± 7.31^c^	56.23 ± 8.02^b^	61.53 ± 6.82^a^

i Note: Different superscript letters (a-c) indicate that there is a significant difference (<0.05). Data presented as mean ± standard deviation.

In the subsequent 56-day adult stage, a reversal in performance trends was observed. Statistical analysis revealed significant differences among treatments for growth parameters (F (6, 54) = 1893.78, *p* < 0.001). While *Schizochytrium* sp. W1.2 initially promoted greater weight gain during the early days of adult culture, *Aurantiochytrium* sp. YA7.1 demonstrated superior overall performance from day 28 through to harvest (see Figure S8 in Supporting Information). This included the best feed conversion ratio (FCR: 1.64, F (3.37, 30.48) = 61.80, *p* < 0.001) and feed conversion efficiency (FCE: 61.5%, F (2.91, 26.18) = 86.70, *p* < 0.001). Moreover, chi-square analysis confirmed that dietary supplementation significantly affected the distribution of shrimp size at harvest (χ^2^(4) = 145.80, *p* < 0.001). Bonferroni post hoc analysis indicated that *Aurantiochytrium* sp. YA7.1 supplementation resulted in a significantly higher proportion of large-sized shrimp (see Figure S9 in Supporting Information) compared to both *Schizochytrium* sp. W1.2 and control groups, while the control group had a higher proportion of smaller individuals. Throughout the entire culturing period, growth trends for both the postlarval (PL) and adult stages displayed significant positive responses to thraustochytrid supplementation, with both linear and quadratic trends (*p* < 0.001). These results reveal clear stage-specific advantages: *Schizochytrium* sp. W1.2 is more advantageous during the early developmental stage, enhancing survival and growth, whereas *Aurantiochytrium* sp. YA7.1 has a greater impact on biomass accumulation and size distribution in adult shrimp.

Importantly, the improved FCR values observed with thraustochytrids supplementation fall within the optimal range of 1.2 to 2.5 reported for shrimp cultivation, indicating not only enhanced growth performance but also better economic viability for shrimp farming.^63^ Collectively, this study highlights the potential of using targeted microbial feed additives from thraustochytrid to enhance survival, growth, and feed efficiency in sustainable shrimp aquaculture.

A 3% inclusion level of thraustochytrid biomass was selected based on prior studies demonstrating its efficacy in enhancing disease resistance while maintaining growth performance.^44^ Using this established supplementation rate, the observed differential performance of thraustochytrid strains across shrimp life stages reflects the distinct nutritional demands associated with development transitions. During the postlarval stage, *L. vannamei* undergoes rapid neural and visual system development, which requires substantial amounts of DHA for the structural integrity and function of cell membranes.^62,64^ Consistent with this requirement, DHA-rich *Schizochytrium* sp. W1.2 significantly improved postlarval survival and length-based growth, supporting its role in early developmental nutrition. Conversely, adult shrimp exhibit different metabolic priorities, where astaxanthin becomes more critical for enhancing stress tolerance and metabolic efficiency. *Aurantiochytrium* sp. YA7.1, characterized by its high astaxanthin content and moderate DHA content, outperformed other treatments in adult shrimp by promoting superior weight gain and feed conversion efficiency. Astaxanthin is a potent antioxidant that enhances stress resistance, improves nutrient utilization, and supports cellular metabolism.^65–67^ Additionally, it has been shown to accelerate molting by increasing ecdysteroid levels and to strengthen immune responses, making it particularly beneficial during stages of biomass accumulation.^68–70^ Collectively, these findings emphasize the importance of life-stage-specific nutritional strategies in shrimp aquaculture. DHA-rich formulations are more suitable during early development for neural and visual tissue formation, whereas astaxanthin-rich supplementation provides advantages during later stages by improving metabolic performance and resilience. Further molecular studies are warranted to elucidate the precise mechanisms underlying these stage-specific effects and to guide the optimization of feeding formulations for different growth phases in *L. vannamei*.

In conclusion, classifying thraustochytrid strains using MALDI-TOF MS Biotyper technology offers improved taxonomic resolution compared to conventional 18S rRNA analysis, particularly for isolates from Taiwan’s mangrove ecosystems. This combined approach integrates proteomic fingerprinting and genotypic analysis, supported by PCA-based K-means clustering, to effectively identify and categorize thraustochytrid strains with potential uses in aquaculture. Among the 26 strains analyzed, biotyper-based identification closely matched with biochemical clustering, revealing four metabolically distinct groups. It is worth noting that *Schizochytrium* sp. W1.2 and *Aurantiochytrium* sp. YA7.1 were identified as optimal candidates for feed supplementation due to their high DHA and astaxanthin content, respectively. Life-stage-specific feeding trials demonstrated that *Schizochytrium* sp. W1.2 significantly improved survival and early growth during the postlarval stage due to its DHA-rich composition, while *Aurantiochytrium* sp. YA7.1 enhanced weight gain and feed conversion efficiency in the adult stage, consistent with the recognized benefits of astaxanthin. These findings establish an efficient pipeline for identifying strains and validating their performance, supporting the use of thraustochytrids as sustainable feed additives in shrimp aquaculture. Moreover, this study offers the aquaculture industry reliable tools for microbial screening, providing an evidence-based alternative to traditional fish oil supplements and enhancing economic efficiency and ecological sustainability in marine farming systems.

## Supplementary Material



## Notes

Source and collection sampling of Formosan thraustochytrid strains, microscopic images of the representative strains, their DHA and astaxanthin contents, information on the growth performance of *Litopenaeus vannamei* in the postlarval stage, as well as their final body weight and size distribution in the adult stage by different addition treatments (PDF)

## ∇

J.-Y.L. is deceased on July 8, 2022.
