# Supplementary material for: MALDI-TOF MS Classification of Formosan Thraustochytrids and Their Use in Shrimp Farming
Source: J Agric Food Chem. 2026 Mar 30;74(13):10747–58. doi: 10.1021/acs.jafc.5c08939 (PMC13067355; doi:10.1021/acs.jafc.5c08939)
Supplement: Supplementary file 1 [file jf5c08939_si_001.pdf]

Supporting Information

MALDI-TOF MS Classification of Formosan Thraustochytrids and Their Use in Shrimp Farming

SETYA WIDI AYUNING PERMANASARI,[†,‡] HSIU-CHIN LIN,[†] JIA-YING LIN,[†] MAO-XUAN HONG,[†] HSIN-YANG CHANG,[†,§] WEN-DI HE,[†,] LI-HUA LO,[†] YU-LIANG YANG,[⊥] TSE-MIN LEE,[†] AND CHIH-CHUANG LIAW[†,||,▽*]

[†] Department of Marine Biotechnology and Resources, National Sun Yat-sen University, 70, Lien-Hai Rd., Kaohsiung 80424, Taiwan, [‡] Department of Fisheries and Marine Resources Management, Fisheries and Marine Science, Brawijaya University, Veteran Rd, Malang 65145, East Java, Indonesia, [§] Department of Life Sciences and Institute of Genome Sciences, National Yang Ming Chiao Tung University, Taipei 11221, Taiwan, [⊥] Agricultural Biotechnology Research Center, Academia Sinica, Taipei 115, Taiwan, [||]Graduate Institute of Natural Products, Kaohsiung Medical University, Kaohsiung 807, Taiwan, [▽] Graduate Institute of Pharmacognosy, Taipei Medical University, Taipei 110, Taiwan

[*]Corresponding author. Tel: +886-7-525-2000, ext. 5058. Fax: +886-7-525-5020. E-mail: ccliaw@mail.nsysu.edu.tw.

[†] Department of Marine Biotechnology and Resources, National Sun Yat-sen University

[‡] Department of Fisheries and Marine Resources Management, Faculty of Fisheries and Marine Science, Brawijaya University

[§] Department of Life Sciences and Institute of Genome Sciences, National Yang-Ming Chiao Tung University

[⊥] Agricultural Biotechnology Research Center, Academia Sinica.

[||] Graduate Institute of Natural Products, Kaohsiung Medical University

[∇] Graduate Institute of Pharmacognosy, Taipei Medical University

List of Supporting Information

Table S1. Source of Formosan thraustochytrid strains.

Table S2. Solvent systems of HPLC qualitative analysis of astaxanthin

Table S3. Comparison of the 18S rRNA phylogenetic tree and the MALDI-TOF MS Biotyper grouping

Figure S1. Collection sampling of Formosan thraustochytrid strains. a. Aogu Wetland (Chia-yi); b. Qigu and Sicao (Tainan); c. Dianbao River (Kaohsiung); d. Shao Chuan Tou (Kaohsiung); e. Love River (Kaohsiung); f. Dapeng Bay (Pingtung); i. Dongsha Island (Dongsha Atoll).

Figure S2. The microscopic images and colony morphologies of representative thraustochytrid strains from each phylogenetic group. Each figure shows a representative strain with consistent imaging: (A) colony edge on agar plate, (B) whole colony morphology, (C) individual vegetative cells at medium magnification, (D) individual cells at high magnification. All cultures were grown on modified GYPA agar plates or in liquid medium for 3 days at 28°C.

Figure S3. Optimization of sample preparation protocol for thraustochytrid MALDI-TOF MS analysis. **Method 1**: vortexing with 100 μL ddH_2O using bead vortexing, the 10 μL supernatant extracted with 50% acetonitrile and sinapic acid (SA) overlay; **Method 2**: extraction with 40 μL SA solution (20 mg/mL in Acetonitrile: ddH_2O (0.1% TFA) 1:1) using bead vortexing; **Method 3**: extraction with 40 μL formic acid using bead vortexing and SA overlay; **Method 4**: vortexing with 100 μL ddH_2O using bead

vortexing, the 10 μL supernatant extracted with 50% trifluoroacetic acid and α-cyano-4-hydroxycinnamic acid solution (12 mg/mL in acetonitrile (0.3% TFA)) overlay.

Figure S4. Time-course optimization of cultivation period for MALDI-TOF MS analysis**.**

Figure S5. Temporal dynamics of biomass accumulation and DHA production in Formosan thraustochytrid strains during batch cultivation.

Figure S6. Temporal dynamics of biomass accumulation and astaxanthin content in Formosan thraustochytrid strains during batch cultivation.

Figure S7. PCA plot analysis based on DHA, astaxanthin, and biomass yield from Formosan thraustochytrid strains.

Figure S8. The trend of growth performance of *Litopenaeus vannamei*, A. body length gain (cm) in the post-larval stage; B. body weight gain (g) in the adult stage.

Figure S9. The final body weight and the size distribution of *Litopenaeus vannamei.*

Table S1. Source of Formosan thraustochytrid strains

No.	Thraustochytrid strains	Species	Collectors
1.	AP10	*Aurantiochytrium* sp.	Dr. Wen-Ming Chen Lab's
2.	AP11	*Aurantiochytrium* sp.	Dr. Wen-Ming Chen Lab's
3.	AP13	*Aurantiochytrium* sp.	Dr. Wen-Ming Chen Lab's
4.	AP15	*Aurantiochytrium* sp.	Dr. Wen-Ming Chen Lab's
5.	AP16	*Aurantiochytrium* sp.	Dr. Wen-Ming Chen Lab's
6.	AP41	*Aurantiochytrium* sp.	Dr. Wen-Ming Chen Lab's
7.	AP45	*Aurantiochytrium* sp.	Dr. Wen-Ming Chen Lab's
8.	YA7.1	*Aurantiochytrium* sp.	Dr. Tse-Ming Lee Lab's
9.	DPB6	*Aurantiochytrium* sp.	Dr. Hsiu-Chin Lin Lab's
10.	SCT01	*Aurantiochytrium* sp. MST3336	Dr. Tse-Ming Lee Lab's
11.	BM2.4	*Aurantiochytrium* sp.	Dr. Tse-Ming Lee Lab's
12.	BM2.5	*Aurantiochytrium* sp.	Dr. Tse-Ming Lee Lab's
13.	SF8.2	*Aurantiochytrium* sp.	Dr. Tse-Ming Lee Lab's
14.	SF8.3	*Aurantiochytrium* sp.	Dr. Tse-Ming Lee Lab's
15.	SF8.5	*Aurantiochytrium* sp.	Dr. Tse-Ming Lee Lab's
16.	TS1.1	*Thraustochytrium* sp.	Dr. Tse-Ming Lee Lab's
17.	TS1.3	*Thraustochytrium* sp.	Dr. Tse-Ming Lee Lab's
18.	TS1.4	*Thraustochytrium* sp.	Dr. Tse-Ming Lee Lab's
19.	TS2.1	*Aurantiochytrium* sp.	Dr. Tse-Ming Lee Lab's
20.	TS2.2	*Aurantiochytrium* sp.	Dr. Tse-Ming Lee Lab's
21.	TS2.3	*Aurantiochytrium* sp.	Dr. Tse-Ming Lee Lab's
22.	TS2.4	*Aurantiochytrium* sp.	Dr. Tse-Ming Lee Lab's
23.	TS3.2	*Ulkenia* sp.	Dr. Hsiu-Chin Lin Lab's
24.	DS15	*Thraustochytrium aff.striatum*	Dr. Hsiu-Chin Lin Lab's
25.	DS18	*Thraustochytrium* sp.	Dr. Hsiu-Chin Lin Lab's
26.	CJ1	*Thraustochytridae* sp.	Dr. Jo-Shu Chang Lab's
27.	UJ1	*Schizochytrium* sp.	Dr. Jo-Shu Chang Lab's
28.	ASP1	*Schizochytrium* sp.	Dr. Jo-Shu Chang Lab's
29.	ASP2	*Parietichytrium* sp.	Dr. Jo-Shu Chang Lab's
30.	ASP3	*Thraustochytrium gaertnerium*	Dr. Jo-Shu Chang Lab's
31.	ASP4	*Botryochytrium radiatum*	Dr. Jo-Shu Chang Lab's
32.	W1.2	*Schizochytrium* sp.	Dr. Hsiu-Chin Lin Lab's
33.	DPBm1	*Schizochytrium* sp.	Dr. Hsiu-Chin Lin Lab's

The nitrogen-dried extract was redissolved in 1 mL of acetone, and insoluble matter was removed by filtration through a 0.22 μm filter. A 20 μL aliquot was withdrawn using a glass syringe and analyzed by HPLC. The analysis conditions for DHA and astaxanthin are presented below.

a. DHA (Docosahexaenoic acid) analysis conditions:

   HPLC analysis was performed using a mobile phase of methanol (A) and ddH2O + 0.1% TFA (B) at a 95:5 (v/v) ratio. The UV detector wavelength was set at 210 nm, and the flow rate was maintained at 1 mL/min. A calibration curve was constructed using a 100 ppm DHA standard solution, with injections of 1, 2, 5, 10, and 20 μL. Peak areas were calculated using HPLC software and processed in Excel. DHA content was quantified using a calibration curve derived from peak area measurements.

b. Astaxanthin analysis conditions:

   The mobile phase conditions were methanol (MeOH), dichloromethane (DCM), acetonitrile (ACN), and 0.1% TFA in deionized water (ddH$_2$O) for gradient elution. The gradient conditions are shown in **Table S2**. Detection was performed at 480 nm, and a flow rate of 1 mL/min was maintained.

   Similarly, the calibration curve was prepared using a 1 ppm astaxanthin standard solution with injection volumes of 1, 2, 5, 10, and 20 μL. Peak areas were integrated using HPLC software, and astaxanthin content was quantified by interpolation from the calibration curve.

Table S2. Solvent system phase conditions for HPLC gradient analysis of astaxanthin

	ACN	MeOH	DCM	DDW
0 min	40	40	16	4
5 min	30	30	36	4
15 min	30	30	36	4
16 min	40	40	16	4
20 min	40	40	16	4

Table S3. Comparison of the 18S rRNA phylogenetic tree and the MALDI-TOF MS Biotyper grouping

18S rRNA phylogenetic	MALDI-TOF MS Biotyper	Strain composition	Genera represented
Clade 1 (19 strains)	Group 2 (4 strains)	DPB6; SCT01; BM2.5, YA7.1	*Aurantiochytrium* sp.
	Group 4 (15 strains)	TS2.2; TS2.1; AP41, AP15; AP10; TS2.3; TS2.4; AP11; AP13; AP16; AP45; BM2.4; TS1.1; TS1.3; TS1.4	*Aurantiochytrium* sp. (TS2.2; TS2.1; AP41, AP15; AP10; TS2.3; TS2.4; AP11; AP13; AP16; AP45; BM2.4) *Thraustochytrium* sp. (TS1.1; TS1.3; TS1.4)
Clade 2 (3 strains)	Group 1 (3 strains)	SF8.3; SF8.2; SF8.5	*Aurantiochytrium* sp.
Clade 3 (4 strains)	Group 3 (4 strains)	ASP1; DPBm1; UJ1; W1.2	*Schizochytrium* sp.
Miscellaneous (7 strains)	Miscellaneous (7 strains)	CJ1; DS18; DS15; ASP2; ASP3; ASP4; TS3.2	*Thraustochytridae* sp. (CJ1)<br>*Thraustochytrium* sp. (DS18)<br>*Thraustochytrium aff. Striatum* (DS15)<br>*Parietichytrium* sp. (ASP2)<br>*Botryochytrium radiatum* (ASP4)<br>*Thraustochytrium gaertnerium* (ASP3)<br>*Ulkenia* sp. (TS3.2)

Figure S1. Collection sampling of Formosan thraustochytrid strains. a. Aogu Wetland (Chia-yi); b. Qigu and Sicao (Tainan); c. Dianbao River (Kaohsiung); d. Shao Chuan Tou (Kaohsiung); e. Love River (Kaohsiung); f. Dapeng Bay (Pingtung); i. Dongsha Island (Dongsha Atoll).

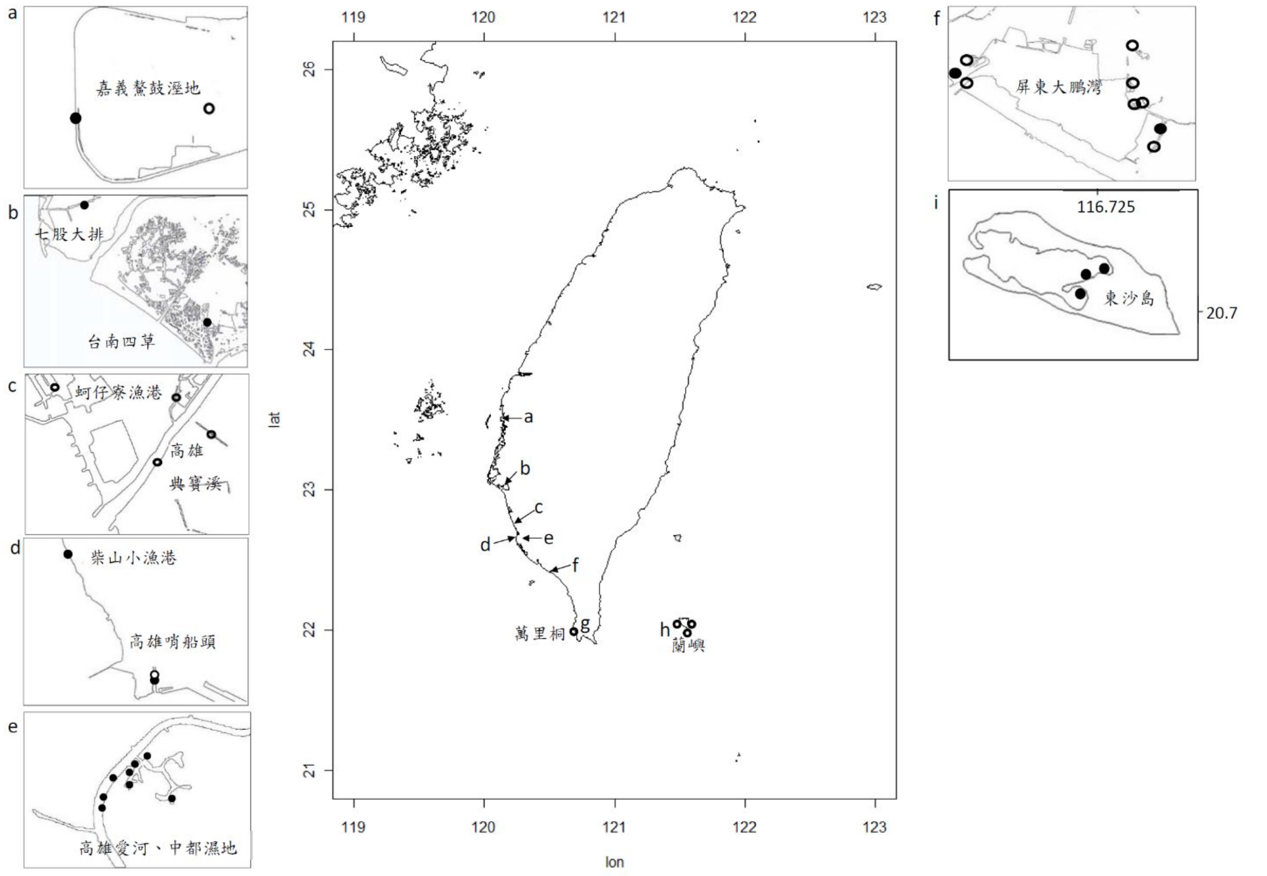

Figure S2. Representative microscopic images and colony morphologies of thraustochytrid strains from each phylogenetic group. Each figure shows a representative strain with consistent imaging: (A) colony edge on agar plate, (B) whole colony morphology, (C) individual vegetative cells at medium magnification, (D) individual cells at high magnification. All cultures were grown on modified GYPA agar plates or in liquid medium for 3 days at 28°C.

a. *Aurantiochytrium* sp. AP11 (phylogenetic **Clade 1**, proteomic **Group 4**)

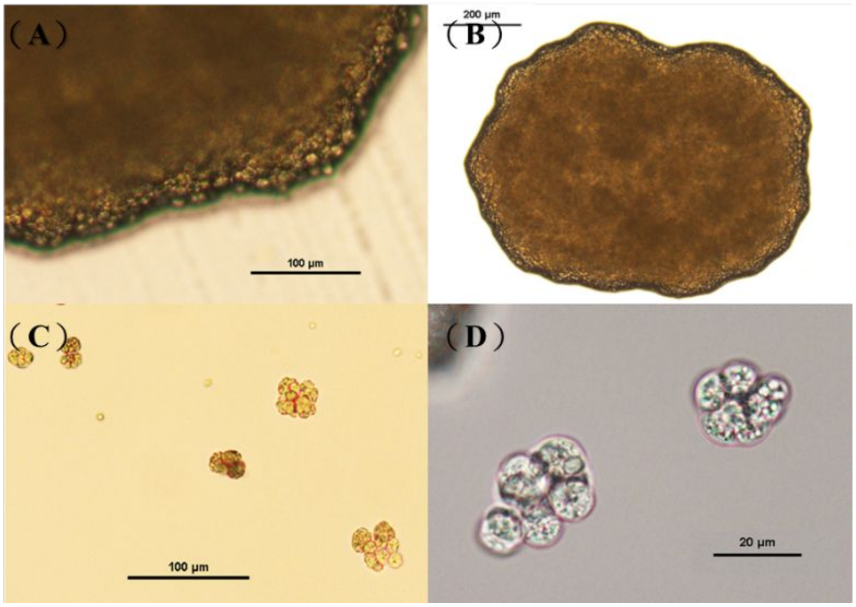

b. *Aurantiochytrium* sp. YA7.1 (phylogenetic **Clade 1**, proteomic **Group 2**)

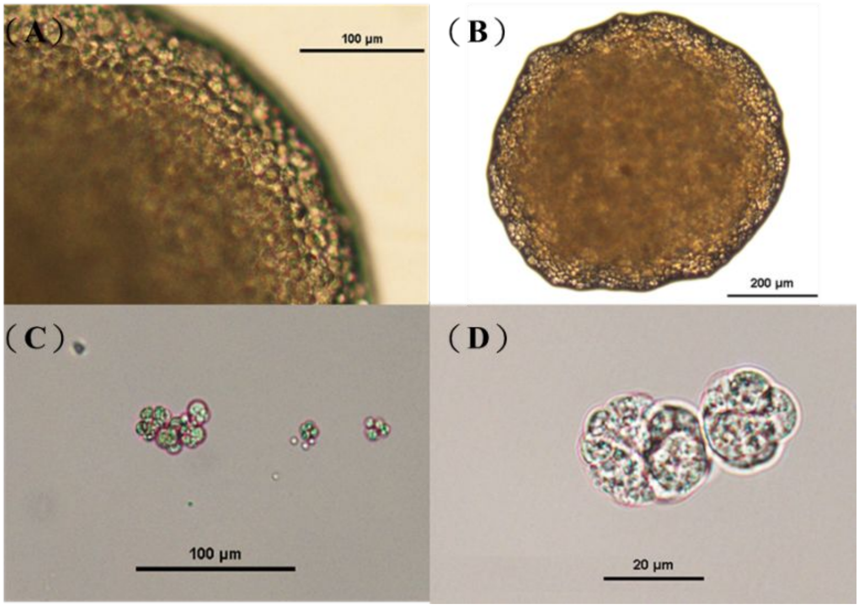

c. *Aurantiochytrium* sp. SF8.5 (phylogenetic **Clade 2**, proteomic **Group 1**)

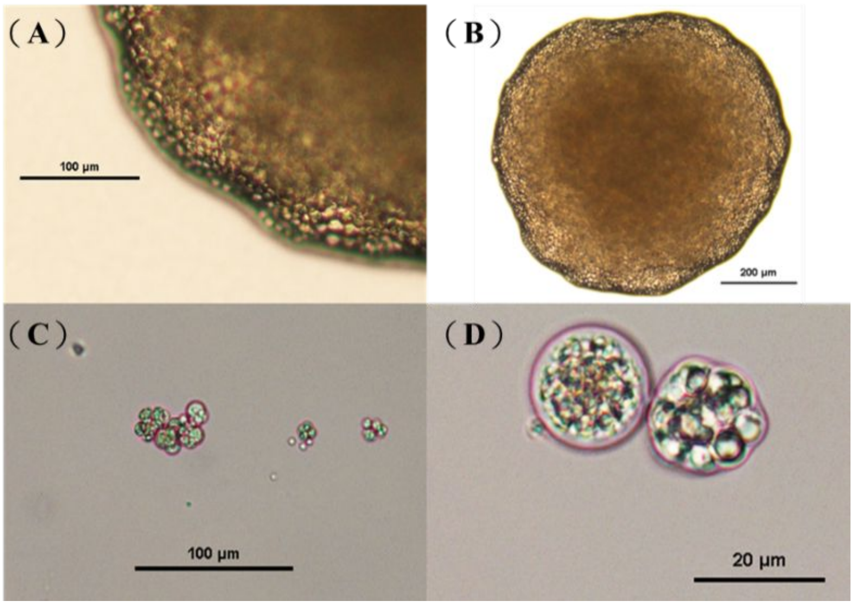

d. *Schizochytrium* sp. W1.2 (phylogenetic **Clade 3**, proteomic **Group 3**)

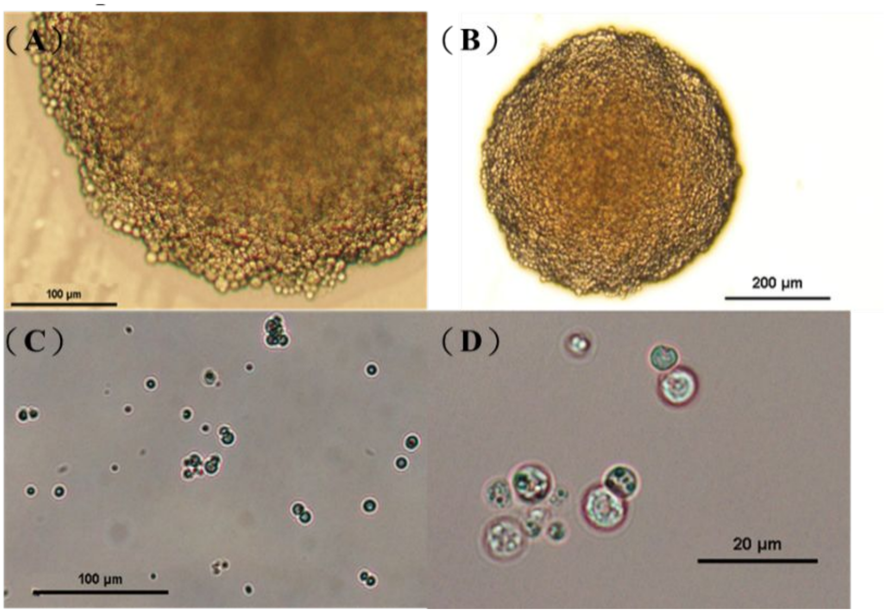

e. *Ulkenia* sp. TS3.2 **(**phylogenetic **Miscellaneous group**, proteomic **Miscellaneous group**)

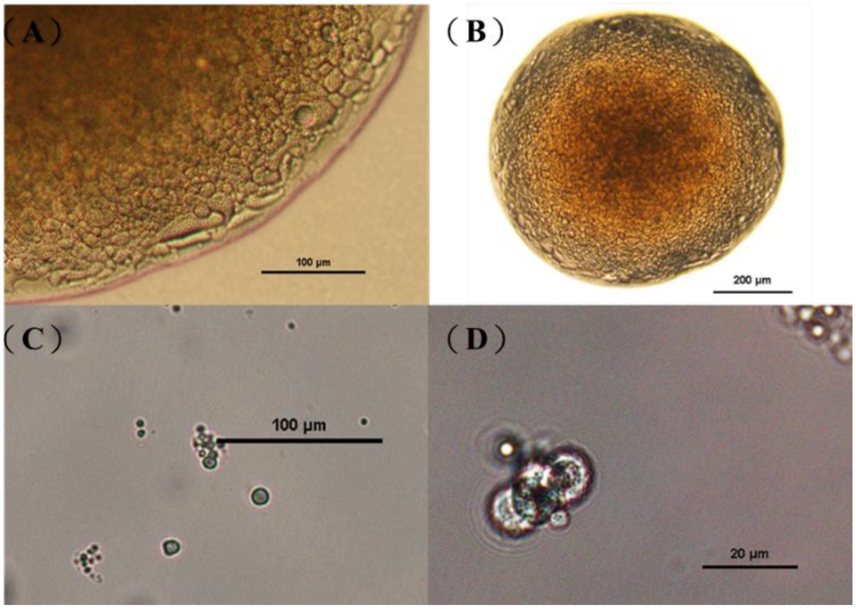

f. *Thraustochytrium* sp. DS18 **(**phylogenetic **Miscellaneous group**, proteomic **Miscellaneous group**)

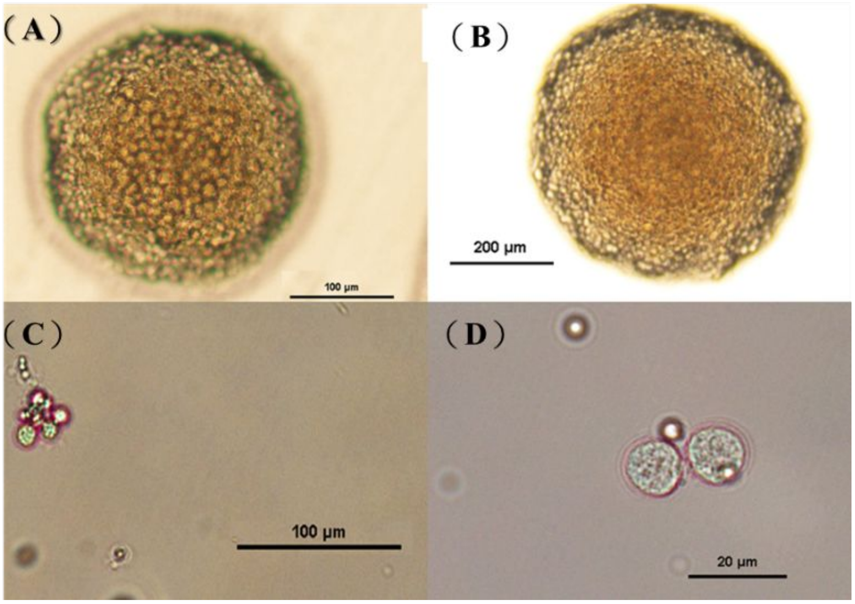

Comparative analysis across rows demonstrates that colony morphology (irregular shapes, smooth margins) and cellular structure (spherical to ovoid cells, 10-30 µm diameter) are remarkably similar across all phylogenetic clades. However, pigmentation intensity provides a clear

distinguishing feature: **Clade 2** displays the most intense pigmentation (dark brownish-orange), **Clade 1** shows moderate orange-yellow coloration, and **Clade 3** exhibits a characteristically pale phenotype with minimal pigmentation. This pigmentation gradient correlates with biochemical analyses: dark-pigmented **Clade 2** strains showed the highest astaxanthin content (proteomic **Group 2**), pale-pigmented **Clade 3** strains showed the highest DHA content with minimal carotenoids (proteomic **Group 3**), and moderately-pigmented **Clade 1** strains displayed diverse proteomic phenotypes (proteomic **Groups 2** and **4**). The morphological uniformity in structural features across phylogenetically and proteomically diverse strains validates the necessity of molecular (18S rRNA) and proteomic (MALDI-TOF MS) approaches for accurate taxonomic discrimination, as traditional morphology-based identification is insufficient for strain-level differentiation in the thraustochytrid collection.

Figure S3. Optimization of sample preparation protocol for thraustochytrid MALDI-TOF MS analysis. **Method 1**: vortexing with 100 µL ddH_2O using bead vortexing, the 10 µL supernatant extracted with 50% acetonitrile and sinapic acid (SA) overlay; **Method 2**: extraction with 40 µL SA solution (20mg/mL in Acetonitrile: ddH_2O (0.1% TFA) 1:1) using bead vortexing; **Method 3**: extraction with 40 µL formic acid using bead vortexing and SA overlay; **Method 4**: vortexing with 100 µL ddH_2O using bead vortexing, the 10 µL supernatant extracted with 50% trifluoroacetic acid and α-cyano-4-hydroxycinnamic acid solution (12 mg/mL in acetonitrile (0.3% TFA)) overlay.

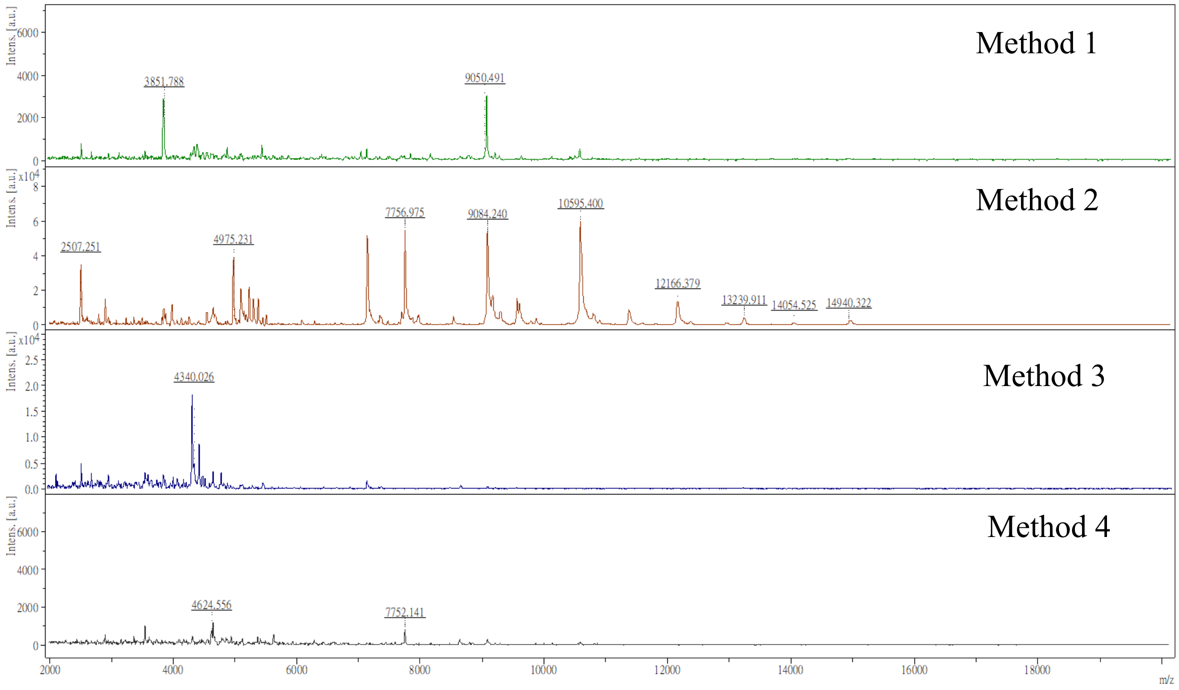

Four extraction protocols were systematically evaluated using *Aurantiochytrium* sp. YA 7.1 to determine the optimal method for thraustochytrid protein profiling: Method 1 (ddH_2O/SA overlay), Method 2 (direct SA extraction), Method 3 (Formic acid/SA overlay), and Method 4 (TFA/CHCA matrix). Method performance was evaluated using three metrics: the number of detected peaks in the *m/z* range of 2,000-20,000, average signal-to-noise ratio, and reproducibility across technical replicates. Method 2 exhibited superior performance, showing higher peak detection and signal intensity than CHCA-based protocols, and therefore selected for subsequent analysis of all 33 thraustochytrid strains.

The superior performance of SA over CHCA likely reflects fundamental differences in cell wall composition between thraustochytrids and bacteria, as CHCA-based protocols are optimized for bacterial identification, whereas SA is better suited for proteins within the target mass range.

During protocol optimization, each spectrum represents the average of five technical replicates, with approximately 500 laser shots per acquisition, accumulated from multiple random positions within each spot. Following method selection, the final protocol employed four replicate spots per strain with five sequential acquisitions per spot (2,000 laser shots per acquisition, collected as four sets of 500 shots at different positions), yielding 20 technical replicate spectra per strain for robust Main Spectrum Profile generation. Error bars represent standard deviation (n=5 technical replicates for optimization). Mass spectra were obtained using an Autoflex Speed MALDI-TOF/TOF mass spectrometer and processed with MALDI Biotyper v3.1 software using the Preprocessing Standard Method.

Figure S4. Time-course optimization of cultivation period for MALDI-TOF MS analysis**.**

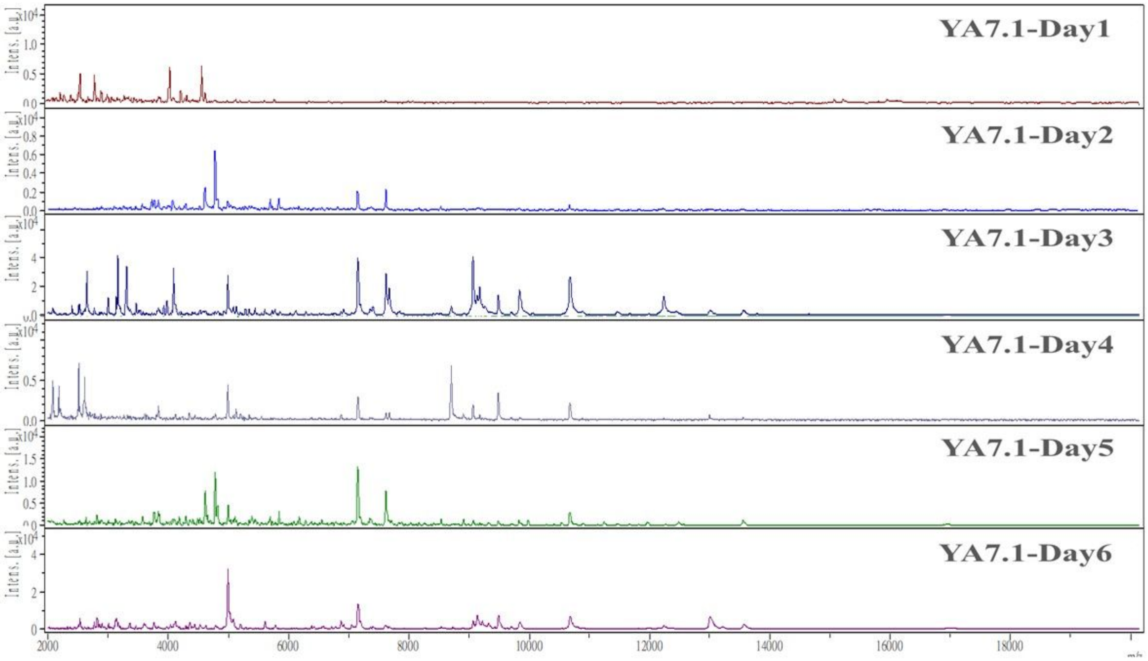

Representative averaged mass spectra of *Aurantiochytrium* sp. YA 7.1 obtained at different cultivation time points (Days 1, 2, 3, 4, 5, and 6) using the optimized sample preparation protocol (Method 2: direct SA extraction). Day 3 cultivation demonstrated optimal spectral quality characterized by:

- Highest overall signal intensity in the *m*/*z* region of 2,000-20,000
- Maximum number of detectable characteristic peaks
- Highest reproducibility across technical replicates (lowest coefficient of variation)
- Distinct proteomic fingerprint pattern suitable for taxonomic discrimination

Based on these optimization results, Day 3 was established as the standard cultivation time point for MALDI-TOF MS analysis of all 33 thraustochytrid strains in this study. MALDI-TOF MS measurements were performed using an Autoflex Speed system (Bruker Daltonics), and spectral data were processed with MALDI Biotyper v3.1 Preprocessing Standard Method.

Figure S5. Temporal dynamics of biomass accumulation and DHA production in Formosan thraustochytrid strains during batch cultivation.

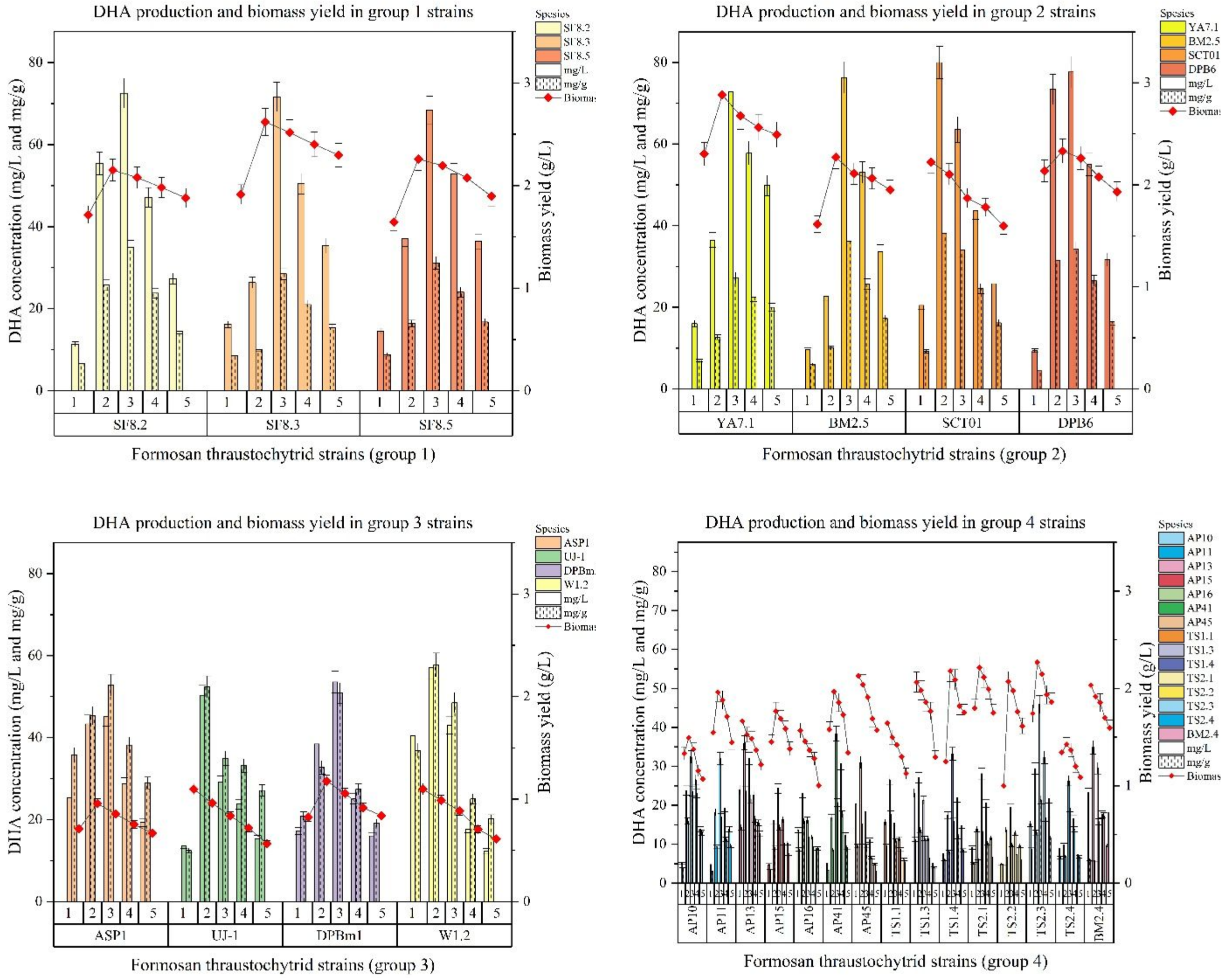

Time-course analysis of dry biomass (mg) and DHA content (mg/g dry biomass) over 6 days of batch cultivation was performed for representative strains from the four MALDI-TOF MS biotyper groups (26 strains total; miscellaneous group excluded due to insufficient biomass). A consistent biphasic pattern was observed across all tested strains, characterized by a peak in biomass preceding the maximum DHA content by approximately 24 hours, with biomass declining as DHA reached its maximum.

Most strains achieved maximum DHA on day 3 following peak biomass on day 2 (Group 1: SF8.2, SF8.3, SF8.5; Group 2: YA7.1, BM2.5, SCT01, DPB6; Group 3: ASP1, DPBm1; Group 4: AP10, AP11, AP15, AP41, TS1.4, TS2.1, TS2.2, TS2.3, TS2.4), while others showed earlier kinetics with day 2 DHA peaks following day 1 biomass peaks (Group 3: UJ1, W1.2; Group 4: AP13, AP16, AP45, TS1.1, TS1.3, BM2.24). Despite these minor temporal variations, all strains exhibited the characteristic pattern of declining biomass concurrent with peak DHA accumulation.

The observed temporal pattern aligns with the biphasic growth physiology of thraustochytrids, characterized by a sequential phase of cellular proliferation followed by biosynthesis.[1] During exponential growth (day 1-2), cultures prioritize biomass accumulation through rapid cell division, with limited intracellular lipid deposition. As nutrient availability becomes limiting and cultures transition toward the stationary phase (day 3 onward), cell replication ceases, and metabolic flux is redirected toward massive lipid accumulation in enlarged, non-dividing cells. The biomass decline observed coincident with peak DHA content likely reflects multiple factors: termination of cell division, partial population lysis, and the energetic trade-off between biomass maintenance and lipid biosynthesis. Early biomass maximum at approximately 48 hours is consistent with previous reports of thraustochytrid batch cultivation.[2]

Data represent mean ± SD from three biological replicates.

Figure S6. Temporal dynamics of biomass accumulation and astaxanthin content in Formosan thraustochytrid strains during batch cultivation.

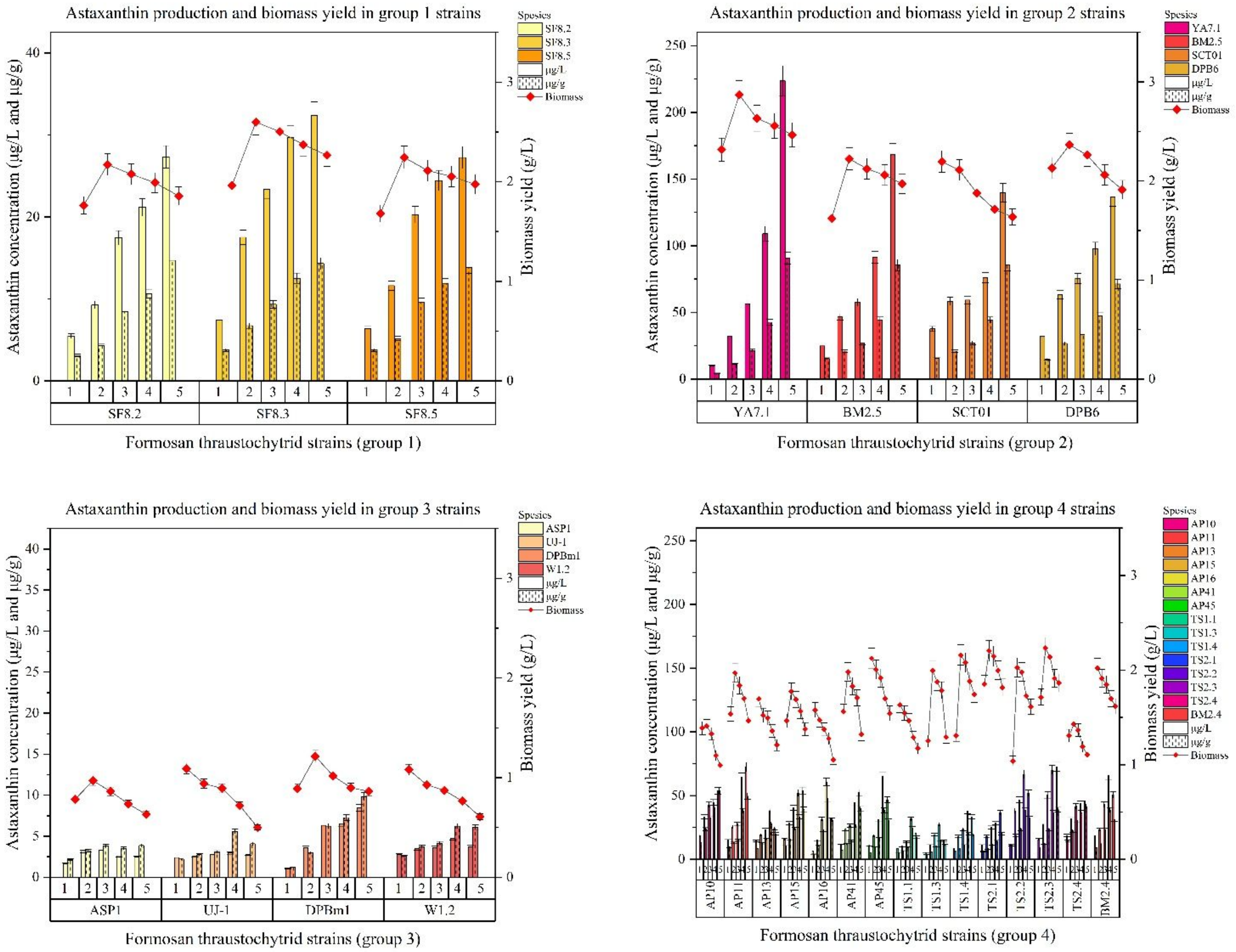

Daily measurements of dry biomass (mg) and astaxanthin content (mg/g dry biomass) over 6 days of heterotrophic batch cultivation for 26 Formosan thraustochytrid strains from four MALDI-TOF MS biotyper groups (miscellaneous group excluded due to insufficient biomass).

Astaxanthin accumulation exhibited a delayed temporal pattern relative to DHA (**Figure S6**), with peak astaxanthin content occurring on days 4-5, approximately 48 hours later than peak DHA content (days 2-3). A similar temporal separation between DHA and astaxanthin biosynthesis, with carotenoid peaks occurring after the lipid accumulation maximum, has been reported previously

in thraustochytrids.[3] The majority strains achieved maximum astaxanthin on day 5 (Group 1: SF8.2, SF8.3, SF8.5; Group 2: YA7.1, BM2.5, SCT01, DPB6; Group 3: ASP1, DPBm1; Group 4: AP10, AP11, AP15, AP41, TS2.1, TS2.3, TS2.4), while others peaked on day 4 (Group 3: UJ1, W1.2; Group 4: AP13, AP16, AP45, TS1.1, TS1.3, TS1.4, TS2.2, BM2.24).

Both DHA and astaxanthin accumulation occurred during the declining biomass phase, with astaxanthin peaking approximately 48 hours after DHA across all strain groups. This temporal pattern was consistent across the 26 tested strains, suggesting that the sequential accumulation of these two metabolites represents a conserved physiological response to batch culture conditions in thraustochytrids. Astaxanthin accumulation was delayed until days 4-5, coinciding with the extended stationary or early decline phase. Similar delayed carotenoid accumulation patterns have been documented in other thraustochytrids and are generally linked to stress responses during late cultivation stages. During this period, nutrient limitation and oxidative stress accumulation are proposed to induce carotenoid biosynthetic genes as a cellular protective against reactive oxygen species.[4]

Data represent mean ± SD from three biological replicates.

Figure S7. PCA plot analysis based on DHA, astaxanthin, and biomass yield from Formosan thraustochytrid strains.

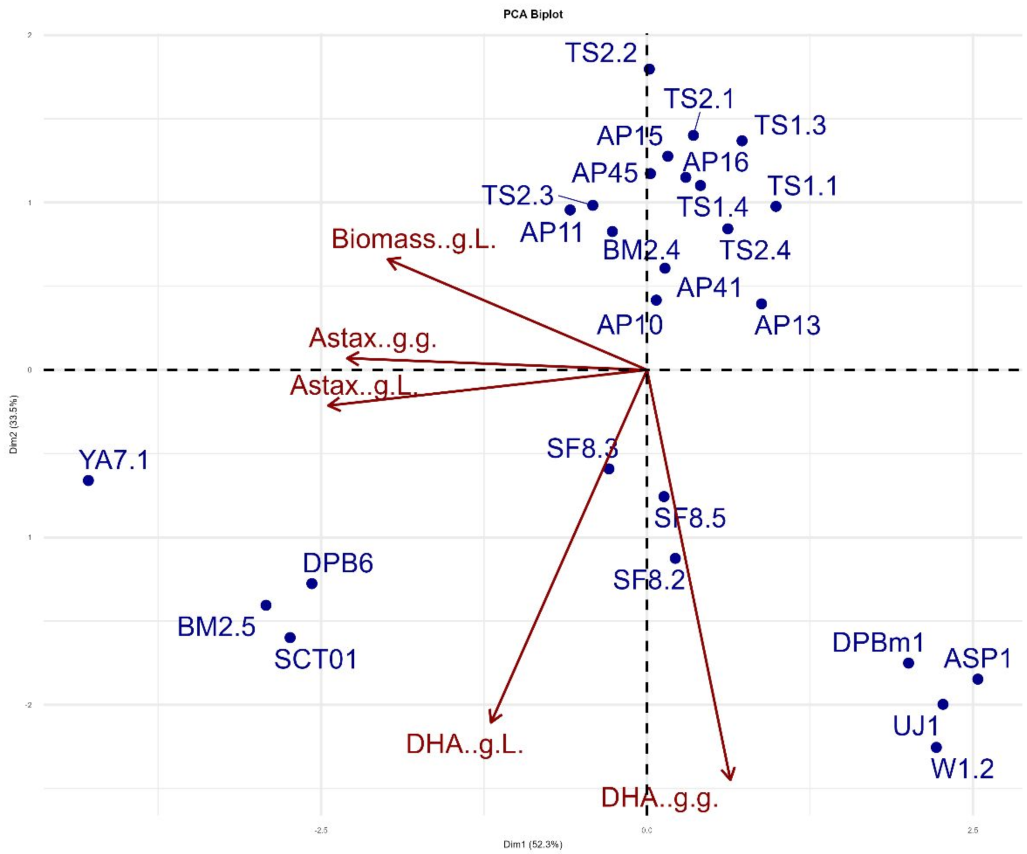

PCA analysis of thraustochytrid strains revealed clear differentiation in DHA and astaxanthin production capacity, with dimension one accounting for 52.3% of the total variance and dimension two contributing an additional 33.5%. This moderate cumulative explained variance (85.8%) suggests that almost all meaningful variation in the data is captured in this two-dimensional projection. The PCA biplot reveals distinct metabolic patterns among the 26 thraustochytrid strains. The loading vectors (red arrows) show the directional relationships among variables, with DHA and astaxanthin displaying opposing patterns along PC1, indicating a fundamental biochemical trade-off between the two compounds. This metabolic specialization is visualized through strain

positioning: high-DHA producers (W1.2, UJ1, DPBm1, ASP1) are grouped in the lower right quadrant, while astaxanthin-rich strains (YA7.1, BM2.5, SCT01, DPB6) are grouped in the lower left quadrant. Strains with intermediate biochemical profiles (SF8.3, SF8.5, SF8.2) position themselves between these extremes, reflecting their balanced production capabilities. The spatial distribution also reflects taxonomic consistency, with *Schizochytrium* species predominantly located in the DHA-rich region and *Aurantiochytrium* species concentrated in the astaxanthin-rich area. PC2 primarily captures variations in biomass yield, providing additional discrimination within similar biochemical profiles. This analysis demonstrates that metabolic specialization corresponds with taxonomic classification and reveals the fundamental trade-off between DHA and astaxanthin production in marine thraustochytrids.

Figure S8. The trend of growth performance of *Litopenaeus vannamei*, A. body length gain (cm) in the post-larval stage; B. body weight gain (g) in the adult stage.

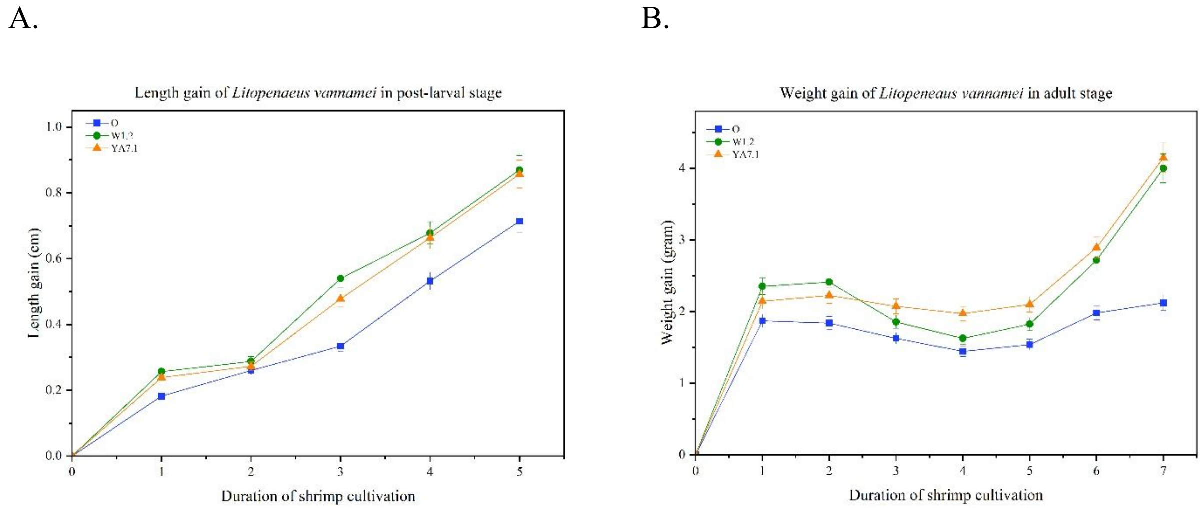

Figure S9. The final body weight and the size distribution of *Litopenaeus vannamei*

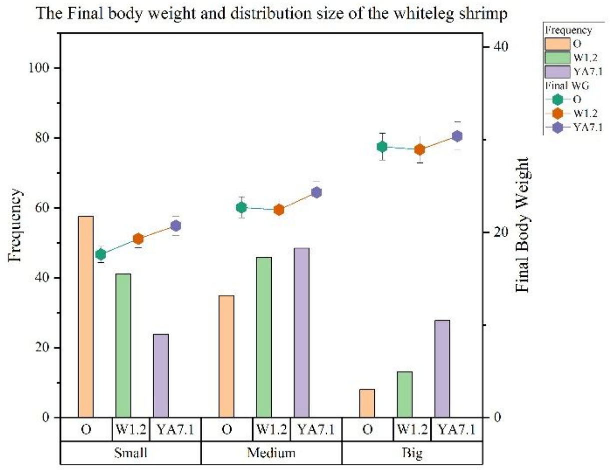

References

(1) Chi, Z.; Liu, Y.; Frear, C.; Chen, S. Study of a two-stage growth of DHA-producing marine algae Schizochytrium limacinum SR21 with shifting dissolved oxygen level. *Appl. Microbiol. Biotechnol.* **2009**, *81* (6), 1141-1148.

(2) Sirirak, K.; Powtongsook, S.; Suanjit, S.; Jaritkhuan, S. Effectiveness of various bioreactors for thraustochytrid culture and production (Aurantiochytruim limacinum BUCHAXM 122). *Peer. J.* **2021**, *9*, e11405.

(3) Quilodrán, B.; Hinzpeter, I.; Hormazabal, E.; Quiroz, A.; Shene, C. Docosahexaenoic acid (C22: 6n− 3, DHA) and astaxanthin production by Thraustochytriidae sp. AS4-A1 a native strain with high similitude to Ulkenia sp.: Evaluation of liquid residues from food industry as nutrient sources. *Enzyme Microb. Technol.* **2010**, *47* (1-2), 24-30.

(4) Aasen, I. M.; Ertesvåg, H.; Heggeset, T. M. B.; Liu, B.; Brautaset, T.; Vadstein, O.; Ellingsen, T. E. Thraustochytrids as production organisms for docosahexaenoic acid (DHA), squalene, and carotenoids. *Appl. Microbiol. Biotechnol.* **2016**, *100* (10), 4309-4321.
